# Reliable identification of homodimers using AlphaFold

**DOI:** 10.1002/pro.70873

**Published:** 2026-11-10

**Authors:** Sarah Narrowe, Arne Elofsson

**Affiliations:** ^1^ Department of Biochemistry and Biophysics and Science for Life Laboratory Stockholm Sweden

**Keywords:** AlphaFold, homodimerization, protein evolution, protein–protein interactions

## Abstract

Protein–protein interactions are central to understanding biological processes. Predicting interaction partners is extremely valuable for avoiding costly, time‐consuming experiments. AlphaFold has shown an unsurpassed ability to predict protein–protein interactions accurately. However, a protein can also form homomeric interactions, that is, interact with itself. When identifying homodimers, AlphaFold yielded a significantly higher false‐positive rate than when identifying heterodimers. True Positive Rate (TPR) at 1% False Positive Rate (FPR) drops from 81% for heterodimers to 22% for homodimers. When we investigated the high‐scoring false positives, that is, non‐homodimers with high AlphaFold scores that were predicted as such, we found that their homologs were enriched for homomultimeric proteins, indicating that stoichiometry is not always conserved among homologs. We split our dataset into a training and test set and with a simple logistic regression model that combines AlphaFold scores with stoichiometry information among homologs and interface features from the predicted structures. This logistic regression model was able to increase the performance on the test set from 27% TPR (at 1% FPR) to 36%. Without homology stoichiometry information, no improvement was seen. The higher number of high‐scoring false predictions for homodimers indicates that homodimer prediction still has significant room for improvement.

## 1 INTRODUCTION

Protein–protein interactions are central to the understanding of biological processes. However, uncovering new interactions is challenging because they range from long‐term, stable complexes to short, unstable, transient interactions. In addition, a protein chain can interact with multiple partners and engage in self‐interactions. Together, these factors make the problem challenging both experimentally and computationally. Not only is it important to know which proteins interact, but it is also important to understand how they interact by uncovering their 3D structures, as protein structure dictates biochemical function (Alberts et al., 2002).

When using AF to predict protein–protein interactions, note that all versions primarily predict a structure rather than an interaction score. This means we must infer an interaction score from the quality of the predicted structure. When Bryant et al. first used AF2.0 for interaction prediction, they developed the pDockQ score for this purpose (Bryant et al., 2022). Later versions of AF include an interaction score, the interface‐predicted TM‐score (ipTM), which should correspond to the quality of the predicted interaction. However, ipTM does not adequately account for disordered regions; therefore, other scores have been developed (Dunbrack, 2025).

Bryant et al. showed that AF2.0 could successfully distinguish interacting from non‐interacting heterodimers (Bryant et al., 2022). Additionally, Schweke et al. showed that by using the predicted aligned error (pAE) together with contact information in the predicted structure, AF2.0 could predict whether a protein forms a biologically feasible homomer (Schweke et al., 2024). In their study, ColabFold (Mirdita et al., 2022) was used, and approximately 45% of the archael and bacterial proteomes and 20% of the proteomes of two eukaryotes were predicted to form homomers.

Two fundamental differences exist between predicting hetero‐ and homo‐dimeric interactions for any method, such as all versions of AF, that is based on co‐evolution. First, to predict heterodimeric interactions, it is necessary to “pair” the two multiple sequence alignments to identify inter‐chain co‐evolutionary signals (Bryant et al., 2022); this is not necessary for homodimers, as they interact with themselves. This should improve homodimer prediction, as pairing often causes information from unpaired paralogs to be ignored.

Secondly, in a homodimeric interactions, co‐evolution occurs for both inter‐ and intra‐chain contacts, and separating these might require structural information of the monomer (Anishchenko et al., 2017). Earlier methods (Pozzati et al., 2022) had problems with this and often produced models in which the two chains overlapped. However, this is generally not a problem for AlphaFold.

Predicting heterodimers also faces a different challenge: homomeric interactions are much more likely than heteromeric ones. The probability that two random proteins form a heteromeric interaction is estimated to be 0.1% (Zhang et al., 2025) while approximately 20%–45% of proteins are expected to form homodimers (Schweke et al., 2024). This makes identifying all heterodimeric interactions more difficult than identifying homodimeric interactions, given the same number of false positives. However, given their abundance, accurate prediction of homodimeric interactions remains important.

In this study, we investigated differences in identifying interacting and non‐interacting protein pairs when the chains are identical (homodimers) versus different (heterodimers). We found that AlphaFold has a lower true‐positive rate (TPR) at low false‐positive rates (1% and 5%) for homodimers than for heterodimers. We sought to understand why and found that the answer may lie in homolog stoichiometry. We observed that stoichiometry varies across all protein classes. Moreover, proteins with many homologs of different stoichiometries are more likely to be mispredicted by AlphaFold. Combining homology and interface information with AlphaFold confidence metrics improved the TPR at a 1% FPR from 27% to 36%. Homolog stoichiometry information was the most important feature. Without this information, the performance dropped to 26%. The best model at 36% still performs worse than for heterodimeric interactions (TPR at a 1% FPR is 81%), indicating that this remains a significant problem.

## 2 METHODS

### 2.1 Dataset generation

We obtained the proteins in the dataset from the Protein Data Bank (PDB) (Westbrook et al., 2002) on June 4, 2026. All of the proteins were released after September 30, 2021, since this is the latest training cut‐off used by any AlphaFold version (Abramson et al., 2024; Evans et al., 2021; Jumper et al., 2021). The collected proteins belonged to one of the three categories: homodimers, heterodimers, and monomers. We filtered the proteins to ensure they were longer than 50 residues and shorter than 2000 residues, contained no DNA/RNA, had a resolution of 4.5 Å or better, and shared less than 90% sequence identity with anything before the training cut‐off. All individual chains in each category were then clustered using MMseqs2 (Steinegger & Söding, 2017) with a minimum sequence identity of 30%, and the center of each cluster was selected. This avoided bias toward a certain class in the test set.

Only heterodimers with less than 90% sequence identity between the two chains were included in the dataset to exclude any pseudo‐homodimers (heterodimers where the two chains are almost identical). We also only used entries containing exactly one bio‐assembly to avoid cases in which different bio‐assemblies in the same PDB entry have different stoichiometries. However, this step excluded many heterodimers, which had the fewest entries. Therefore, for heterodimeric entries, we allowed multiple bio‐assemblies, provided all assemblies had the same stoichiometry. The data selection process is visualized in Figure 1 and is explained in detail in the Supplementary material Section S3.1 and Tables S11–S16 show how many proteins were excluded in each step of the filtering process.

**FIGURE 1:**
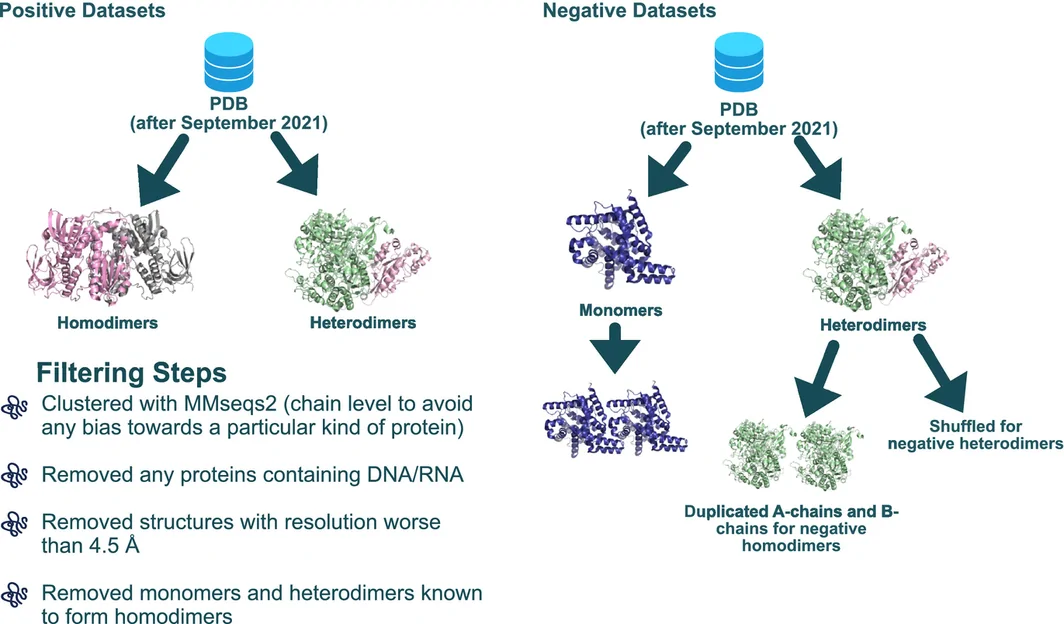
An overview of the dataset generation process.

To ensure the monomeric dataset did not contain any sequences found in multimers, we removed monomers that shared 90% or more sequence identity with any multimer. We identified these monomers with MMseqs2 using the easy‐cluster method with a 0.9 threshold, and removed any monomers in the same cluster as a multimer. The same process was also repeated for heterodimers, resulting in the exclusion of any heterodimers that appeared in the same clusters as homodimers. The final PDB IDs used in the study are available in Tables S1–S5.

After all filtering, the dataset consisted of 783 monomers and 716 homodimers. A total of 139 complete heterodimers passed the initial checks. When testing AF's prediction of heterodimeric interactions, all 139 pairs were included. However, we excluded 20 of these from the negative homodimer set because both chains also exist as homodimers. Therefore, 119 were used as negatives, corresponding to a total of 238 individual entries. Additionally, 346 individual heterodimeric chains that passed all filters were also used as negatives. This led to a total of 584 heterodimeric entries. This yielded 2083 individual entries in the homodimeric dataset.

#### 2.1.1 Negative datasets

As a negative control for homodimeric predictions, we used the 783 monomers and 584 individual chains from the heterodimers. We treated each chain individually and predicted it as a homodimer. For heterodimers, we drew inspiration from the construction used in RoseTTAfold‐PPI (Zhang et al., 2025). Using the monomeric and heterodimeric entries, pairs of individual chains were selected randomly. Each pair was checked against the STRING database (Szklarczyk et al., 2023) to ensure that no interacting proteins were included as negatives. We kept as many same‐species negative interactions as possible to create a more realistic scenario. To keep the negative‐to‐positive ratio the same for homodimers and heterodimers, we used 293 negative chains, maintaining an approximate 2:1 ratio. For details, see the Supplementary Materials, Section S3.1.6.

### 2.2 AlphaFold settings

In this study, we assessed the performance of AlphaFold (AF) 2.0, 2.3, and 3.0. The multiple sequence alignments (MSAs) used by all AlphaFold versions were created with MMseqs2 (Steinegger & Söding, 2017) using the default settings of ColabFold (Mirdita et al., 2022). Additionally, we modified the MSA settings for AF2.3 to investigate their effect on performance on the homodimeric datasets. We ran all methods with default settings, without templates. However, across all methods, we generated only 5 structures, not 25, the default for AF2.3 and 3.0. Each AF2 version comprises five distinct models with different training regimens, and we predicted a structure for each model. For AF3, we generated five models using random seeds 1–5.

We calculated the maximum, minimum, and average of ipTM, pTM, and ranking_confidence (for AF2.3 and AF3) across the five models. The ipTM calculation for AF2.0 is not present in the original AF2.0; instead, ColabFold (Mirdita et al., 2022) implements it.

We tested running AF2.0 with both the FoldDock (Bryant et al., 2022) and ColabFold (Mirdita et al., 2022) pipelines. It was observed that the ColabFold pipeline performed significantly better on the homodimeric dataset, producing fewer instances of complete chain overlap. This is likely because ColabFold does not pair sequences in the MSA for homo‐oligomers and instead uses gapped alignments. Therefore, we ran AF2.0 only with ColabFold. We also used ColabFold to predict structures as input to the logistic regression model QSproteome (Schweke et al., 2024), which uses one structure per AF2.0 model (i.e., 5 predictions).

#### 2.2.1 Identification of interacting and non‐interacting pairs

The quality of an AF model can be estimated from internal scores, including ipTM or Ranking Confidence, as well as from external methods that evaluate the model's structure. These metrics can be assessed for a single prediction, or multiple predictions of the same protein complex can be used to obtain the minimum, maximum, or average. When assessing the performance of the AF models in this study, we focused on the highest (max) ranking confidence for each protein complex, since it is common practice to use the highest‐ranked structure.

### 2.3 Conservation of stoichiometry of homologs

We used the structure‐based search tool Foldseek (Kempen et al., 2023) to query proteins in our dataset against the PDB (updated on the June 4, 2026) to investigate the stoichiometry of structurally similar proteins. We assume that most hits with high TM‐scores are homologs, whereas lower TM‐scores may include non‐homologs due to convergent evolution. For the search, we used Foldseek in monomer mode, that is, individual chains in both the positive and negative datasets were compared with the rest of the PDB using experimental structures. Additionally, MMseqs2 (Steinegger & Söding, 2017) was used to search the same dataset using only sequence information.

All homologs detected by Foldseek (or MMseqs2) were classified into three stoichiometry categories: monomers, homomultimers, and heteromultimers, based on the number and type of chains in the biological units. We computed the fraction for each category at TM‐score cutoffs from 0 to 0.999 in 0.001 increments. Queries lacking hits at the current cut‐off were excluded from the calculations. For each query structure, we filtered Foldseek hits at a given TM‐score threshold and deduplicated them so chain variants of the same assembly (e.g., chains A and B of the same biological unit) contributed only once, retaining the highest TM‐score. If the same PDB code had multiple assemblies, we normalized them. This avoided the scenario where a single PDB code with many bio‐assemblies would outweigh entries with few assemblies. We computed the fraction of each stoichiometry category as the weighted count divided by the total number of unique target proteins passing the threshold. We excluded self‐hits (targets sharing the query's PDB code) throughout.

### 2.4 Logistic regression

Because AlphaFold is less accurate at identifying homodimers than heterodimers, we examined whether we could improve performance by incorporating predicted‐structure information into a machine learning method. We started by identifying features to include, and then we selected the best features and combined them using a logistic regression function.

#### 2.4.1 Features for improved homodimer identification

Inspired by the structure prediction and omics‐informed classifier (SPOC) presented in Schmid and Walter (2025), we extracted features from the predicted complexes and their interfaces. Additionally, we used various versions of the AlphaFold confidence metrics, along with stoichiometry information among homologs. After identifying the best feature set using multiple strategies from the scikit‐learn library (Pedregosa et al., 2011), we used a simple logistic regression model to improve homodimer prediction. The optimization focused on improving the TPR at 1% FPR and the area under the precision‐recall curve (AUPR).

##### Homolog stoichiometry information

Previous studies (Dey et al., 2018; Xu & Dunbrack, 2011) have shown that investigating whether the homodimer of interest contains a conserved interface provides strong evidence for predicting functional homodimeric interfaces. Therefore, we searched the PDB using the predicted structure to identify proteins with similar structures and examined their stoichiometries. However, when building a predictor of homodimer formation, it is crucial to avoid overfitting to proteins with strong homology signals (i.e., proteins with an almost identical sequence to the query). In many cases, this would effectively provide the answer to the model. Therefore, MMseqs2 was used to retrieve the *F‐ident* (approximate sequence identity) between the Foldseek matches and the query. Any Foldseek matches with *F‐ident* above 0.5 to the query were removed. From the remaining Foldseek hits, fractions of homomultimers (*HM Fraction*) and fractions of all multimers (*Multimer Fraction*) were derived at TM‐score cut‐offs ranging from 0.0 to 0.9 in increments of 0.1.

For the logistic regression, we also engineered additional homology features that should, in theory, provide more information about the presence of homologs:

- hm_minus_multimer_tm08: Homomultimer minus multimer fraction at TM‐score cut‐off 0.8. This feature captures whether structures with TM‐score above 0.8 are more homomultimer‐like or heteromultimer‐like.
- hm_high_any: This is a binary 0 or 1 feature that is intended to indicate whether a pair has any high‐scoring (TM‐score above 0.8) homomultimer at all.
- hm_low_high_gap: This feature is calculated by taking the homomultimer fraction at TM‐score 0.2 minus at TM‐score 0.8. The hypothesis is that this feature can capture the shape of the hit‐quality distribution rather than a single threshold.
- foldseek_specificity: Homomultimer fraction at TM‐score 0.8 divided by the multimer fraction at TM‐score 0.8 (with a small value added to avoid division by zero). This feature has the same intent as (hm_minus_multimer_tm08) but should instead capture the information as a ratio rather than a raw difference.

When there were no hits (either from the start or after the MMseqs2 filter), the fractions were set to −1. The fraction was set to 0.0 if the search yielded matches that were neither homomultimers nor multimers. We also retrieved the highest TM‐score for homomultimers, multimers, and all matches. If a match was missing, we imputed the value with −1. In the end, a total of 23 features summarizing the dataset's homology were included in training the logistic regression model. For the individual performance of each feature, see Supplementary Table S6. When retrieving these features, Foldseek was used in TM‐align mode (which normalizes the score by the length of the query), and the targets were pooled and deduplicated in a similar fashion when collecting stoichiometry information for the dataset, as explained in Section 2.3.

##### AlphaFold confidence metrics

From the standard AlphaFold metrics, ipTM, pTM, and ranking confidence were collected for each of the 5 predicted structures. We then collected the minimum, maximum, and average for each query. In addition to the standard AlphaFold metrics, we also included min, max, and mean ipSAE (Dunbrack, 2025)‐values for each query.

In addition to standard AlphaFold confidence metrics, we engineered derived features that capture how consistently the predicted models agreed and how far related confidence metrics diverged, to examine whether this could improve predictive performance; see Supplementary Table S6. These features were the following:

- iptm_spread: Average minus minimum ipTM score across all predictions. This feature captures how much the predicted models disagree about interface quality. A large spread indicates an unstable, less reproducible prediction, while a small spread indicates that the models consistently agree.
- rc_spread: Average minus minimum ranking confidence across the set of predictions. This feature has the same intent as iptm_spread, but is applied to ranking confidence rather than ipTM.
- iptm_rc_prod: Minimum ipTM multiplied by minimum ranking confidence. The hypothesis is that this feature captures predictions where both metrics are jointly low (or jointly high) in their worst case, rather than treating a poor worst‐case ipTM and a poor worst‐case ranking confidence as independent signals.
- ipsae_iptm_diff: Average ipSAE minus average ipTM. The intent behind this feature is that it should capture cases where these two related interface‐confidence metrics disagree, which can happen in cases with a high amount of disorder and accessory domains

##### Structural consensus

Previous work in Fromm et al. (2026) has noted that when AlphaFold predicts correctly, the predicted structures are often very similar. In contrast, when a prediction is wrong, they are often different. Therefore, USAlign (Zhang et al., 2022) (with the flag‐mm 1) was used to calculate the similarity between all 5 predicted structures of one target (excluding self‐comparisons and symmetric comparisons), resulting in $\frac{5^{2}-5}{2}$ comparisons. Since the query and target lengths were identical for all searches, it did not matter whether the score was normalized by the query or target; hence, we always used the first TM‐score. We then included the minimum, maximum, and mean TM‐scores for these comparisons as features, called structural_consensus_min/max/mean.

##### Properties of interface surface

When gathering features for SPOC (Schmid & Walter, 2025), several clever ways were used to characterize contacts and pairs of residues in the interface of a predicted protein–protein structure. In the original SPOC model, 42 features were included and later pruned to 24 using the Gini importance from their random forest model. Of these 24, we also include the 12 structural features in our feature set. The remaining 12 features come from genetic database information and AlphaMissense (Cheng et al., 2023) features, which are only available for human proteins and are therefore not usable for general predictions.

The 12 structural features include *min contacts across predictions*, *num contacts with max n models*, *num unique contacts*, *mean contacts across predictions*, *best num residue contacts*, *best if residues*, *best pLDDT max*, *best contact score max* and *best pAE min*. These 12 features also include the minimum, maximum, and average ipTM values as mentioned in Section 2.4.1. For an explanation of each feature, see Table S7 and the Predictomes paper (Schmid & Walter, 2025).

Next, to analyze the polarity and size of the interfacial surface, FreeSASA (Mitternacht, 2016) was used to calculate the Solvent Accessible Surface Area (SASA). From the output, we retrieved the a/polar relative and raw interface sizes, as well as the total interaction area. For each protein pair, the minimum, maximum, and average of these values across the five predicted structures were retrieved as inputs to the logistic regression.

#### 2.4.2 Feature selection

In total, 70 features were collected (24 interface/structural features, 27 homology‐based features and 19 from AlphaFold (including 3 from ipSAE)) to serve as input to a logistic regression model; see Table S6. However, we cannot guarantee that all of these features improve homodimer identification. Testing all combinations of these 70 features would have been ideal to find the optimal solution. In practice, however, this is infeasible because it would require testing 70! ($1.2\times 10^{100}$) combinations. Therefore, we employed six complementary strategies to identify the best feature combinations (see Supplementary Material Section S3.3.2 for details). We selected the best one based on average performance on the training set using a stratified 5‐fold cross‐validation. We selected the feature set with the best average AUPR and the best TPR at 1%FPR.

This feature selection procedure was repeated twice: once including all homology features and once excluding them. The final performance was reported on the test set, which was not used for any optimization or feature selection. We randomly split the data into training/test sets at a 60/40 ratio, maintaining the same negative‐to‐positive ratio in each split.

#### 2.4.3 Fitting a logistic regression function to the features

After identifying the best set of features, we fitted a logistic regression model to the entire training set and tested it on the test set. Logistic regression models the probability of a positive outcome by passing a linear combination of input features through a sigmoid function:

$$P\left(y=1|x\right)=\frac{1}{1+e^{-\left(\beta_{0}+\beta^{⊤}x\right)}}$$

where the coefficients β are learned by maximizing the log‐likelihood on the training data.

## 3 RESULTS AND DISCUSSION

Previous work has demonstrated the high performance of AlphaFold (AF2.0 and AF2.3) in distinguishing interacting from non‐interacting heterodimeric pairs (Bryant et al., 2022; Martin, 2024; Zhang et al., 2025). AF2.0 can also distinguish physiological from non‐physiological homodimeric interfaces (Schweke et al., 2024). However, to the best of our knowledge, it is not well established whether AF2.3 and AF3.0 can accurately identify homodimers. This study aims to address this question.

### 3.1 High scoring false positives for homodimers

First, AF2.3's ability to separate interacting and non‐interacting protein pairs was assessed. A major challenge in this study was defining a reliable negative dataset for homodimer prediction, as it is difficult to confirm that a protein does not form a homodimer under any condition.

Another concern is that the ground‐truth stoichiometry of the proteins in the dataset was inferred from the bio‐assemblies in the PDB.*cif* files, with the understanding that these files may contain errors. To minimize the impact of potential classification errors in the negative dataset, we used two different negative sets. The first dataset consisted of monomers and the second of separated heterodimeric chains (see Section 2.1). AF2.3 achieved better performance when using heterodimers as negatives across all included metrics, with the most noticeable differences in true positive rate (TPR) at false positive rates (FPRs) of 1% and 5% (see Table 1). However, all of the negative datasets, separately and combined, perform significantly worse than on the heterodimer dataset. In Table 1, all metrics are normalized for a 2:1 negative:positive ratio.

**TABLE 1:** Area under the precision–recall curve (AUPR), F1 score, and MCC score.

Dataset	AUPR (↑)	F1 (↑)	MCC (↑)	TPR@5%FPR (↑)	TPR@1%FPR (↑)
All homodimers	0.78	0.72	0.61	0.59	0.22
Neg: monomers	0.77	0.71	0.59	0.54	0.20
Neg: Dup. Hete Chain	0.81	0.74	0.64	0.63	0.31
Heterodimers	**0.93**	**0.86**	**0.81**	**0.86**	**0.81**

*Note*: F1‐score and MCC‐score are calculated using the highest‐ranking confidence for each PDBid, with a threshold of 0.8. The best value for each metric is marked in bold. All values are normalized to a 2:1 ratio for positives and negatives.

Upon examination of the precision‐recall curves in Figure 2a and the area under the precision–recall curve (AUPR) in Table 1, AF2.3 clearly maintains a higher precision for heterodimers than homodimers. AF2.3 is also able to correctly label more positives at low FPRs for heterodimers, as seen by analyzing the raw TPR values at the low FPR of 1%, which is 81% for heterodimers versus 22% for the combined homodimer set. This can be explained by a significantly higher number of high‐scoring negatives in both negative homodimeric datasets; see Figure 2b, where negatives scoring above 0.8 are absent for heterodimers. Figure 2c shows the score distribution for the positives in the dataset, which is similar for both heterodimers and homodimers. The performance on the homodimeric dataset is similar across all versions of AlphaFold and QSProteome. Therefore, for the remainder of this study, the focus is on the results of AF2.3, while the results of the other AlphaFold versions and QSProteome can be found in Table S8.

**FIGURE 2:**
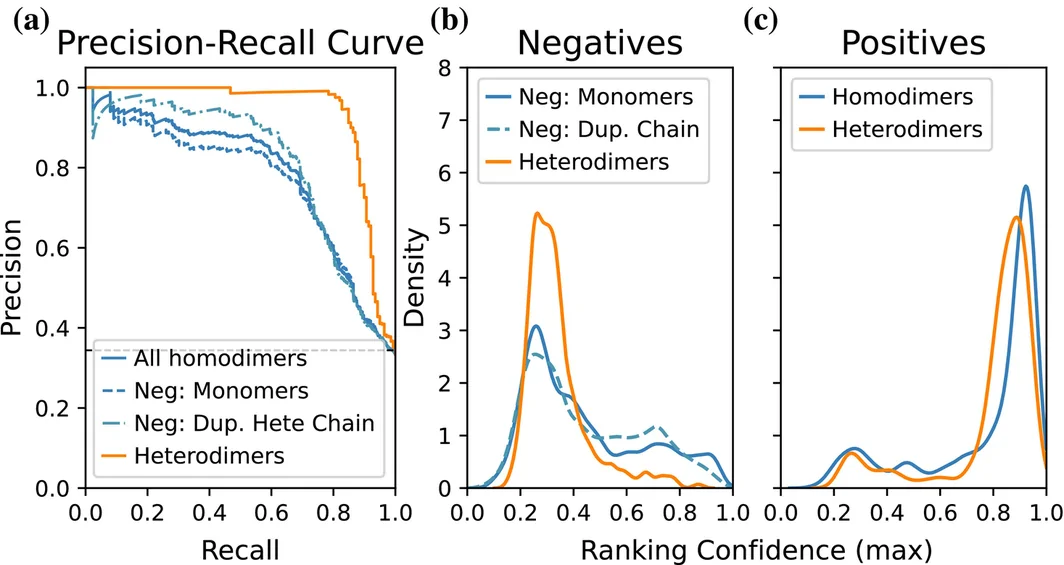
The results of AlphaFold predictions of homodimerization vs. heterodimerization. (a) Precision‐recall curves for heterodimers and homodimers (with monomers and heterodimers used as negative datasets). (b) Density plots for the negative datasets. (c) Density plots for the positive datasets.

For large‐scale prediction studies, heteromeric and homomeric predictions face different challenges. Assuming that each protein, on average, only interacts with a few other proteins (which is true for the vast majority of proteins), the probability that two different proteins interact is less than 0.1% (depending on the size of the proteome), while the probability that it interacts with itself has been estimated to be 20%–45% (Schweke et al., 2024; Zhang et al., 2025). Therefore, we calculated the AUPR at signal‐to‐noise ratios of 1:4, 1:10, 1:100, and 1:1000 (by reweighting the negative samples) for homodimers and heterodimers, see Table S9. Performance is better for heterodimers than for homodimers at each ratio. However, when considering realistic ratios with homodimers at a 1:4 (Schweke et al., 2024) and heterodimers at a 1:1000 (Zhang et al., 2025), performance is better for homodimers, AUPR 0.68 versus 0.52, indicating that there is a need for improved predictions both for homomers and heteromers.

### 3.2 Difference between prediction of homo‐ and hetero‐dimers

Next, we sought to find the underlying reasons for the high‐scoring negatives.

AlphaFold uses multiple sequence alignments to predict protein structures. Therefore, one possible reason for the high false‐positive rate might be the presence of homodimers (or homomultimers) within the MSA. To test whether a stricter *E*‐value cutoff, fewer iterations, or focusing on orthologs could reduce false positives, we regenerated the MSAs using stricter *E*‐value cutoffs, one iteration, and only the top‐scoring homolog in each species; see Table 2. Including only the top‐scoring sequence for each species increased AUPR from 0.78 to 0.79, F1‐score from 0.72 to 0.74, and MCC from 0.61 to 0.62, using the prediction with the highest ranking confidence (RC) and a threshold of 0.8. However, TPRs at 1% and 5% remain unchanged when compared to the default settings. Using the strict *E*‐value of $10^{-60}$ decreased all metrics, indicating that this strict setting is not optimal for homodimer prediction. This small experiment suggests that MSA settings are not the primary reason for AF2.3's reduced homodimer prediction performance.

**TABLE 2:** Area under the precision‐recall curve (AUPR), F1‐score and MCC‐score for the different MSA settings.

MSA setting	AUPR (↑)	F1 (↑)	MCC (↑)	TPR@5%FPR (↑)	TPR@1%FPR (↑)
*E*‐value = 10^−1^, 3 iterations (Default)	0.78	0.72	0.61	0.59	0.22
*E*‐value = 10^−60^, 1 iteration	0.61	0.43	0.40	0.36	0.21
Orthologs (1 Seq Per TaxID), 3 iterations	0.79	0.74	0.62	0.59	0.22

*Note*: The thresholds for F1 and MCC are 0.8. All measured targets are scored based on the maximum ranking confidence across all five generated models.

### 3.3 How conserved is stoichiometry?

Since filtering the MSA content did not affect performance, we theorize that, to interpret the results of AF2.3, it is important to assess how well stoichiometry is conserved, as described in Section 2.3. When displaying the results, three classes of stoichiometry were defined: monomers, homomultimers, and heteromultimers, and their fractions were calculated; the results are shown in Figure 3a–c.

**FIGURE 3:**
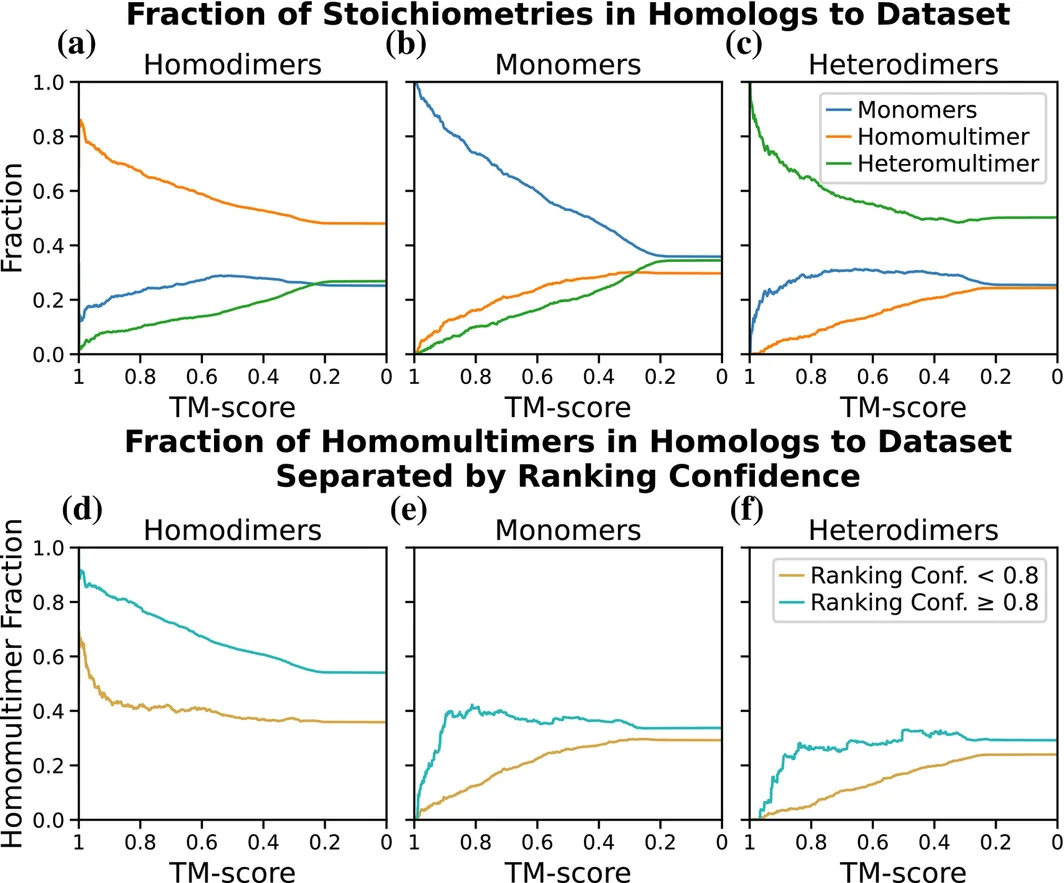
Fractions of stoichiometries of homologs at different TM‐score cut‐offs. (a)–(c) The stoichiometry fractions of the three datasets: homodimers, monomers and negative heterodimers. (d)–(f) The homomultimer fraction for the homologs of the three datasets, divided into predictions with ranking confidence above or below 0.8.

The results show that the filtering of monomers and heterodimers was successful. Inferring the raw values for matches observed at TM‐score 0.999 or higher shows that no multimers are present among monomers and no homomultimers among heterodimers. For heterodimers, very few monomers are also present (0.8% in total). However, it is important to note that these observations were made with a small sample; at the 0.999 threshold, only 58 homodimers, 47 monomers, and 22 heterodimers had any hits at all (excluding self‐hits). We did not use MMseqs2 to filter homodimers against entries with other stoichiometries, as we did for monomers and heteromultimers, so this category remains more diverse. We reasoned that homodimers that meet the criteria for inclusion in the positive set should not be excluded solely because the same sequence is present in entries with different stoichiometries. In contrast, this filtering was important for the negative dataset to reduce the risk of including potential homodimers among the negative examples. Consequently, there are 11.3% monomers and 1.7% heteromultimers present with TM‐scores equal to or above 0.999 for homodimers; see Table S10. Upon manual inspection, many high‐scoring hits with different stoichiometries may be unreliable annotations in the PDB; however, manually investigating all hits is beyond the scope of this study.

Across all classes, as TM‐score cutoffs decrease, the diversity of similar structures increases. When considering only TM‐scores of 0.2 or lower, the fractions of homomultimers for homodimers and heteromultimers for heterodimers both drop to about 50%. Meanwhile, the percentage of monomers drops to as low as 35%. This could explain why AF2.3 struggles more with monomers as negatives than with heterodimers.

In Figure 3d–f, the stoichiometry fractions have been separated for proteins predicted with maximum ranking confidence 0.8. This separation clearly shows that pairs predicted with ranking confidence of at least 0.8 always have a higher fraction of homomultimers. The fraction is slightly higher for monomers than heterodimers, which may explain why the TPR for heterodimers is better at the low FPR rates of 1% and 5% as observed in Table 1. However, between ranking confidence levels of 0.6 and 0.8, there are slightly more monomers; see Figure 2b, indicating that this difference might be smaller if a ranking confidence cut‐off of 0.6 was used.

Extending the distributions of the negative samples above 0.8 to all three stoichiometry classes in Figure S2e,f,h–i in the Supplementary Material strengthens this observation. This is especially clear for the monomeric dataset, which further explains why AF2.3 performed worse on these negatives. Comparing Figure S2d,g reveals that the monomeric fraction of the positive samples is substantially increased for false negatives, and the homomultimeric fraction is simultaneously decreased.

When analyzing sequence similarity rather than structural similarity with MMseqs2, the results are somewhat similar but show slightly higher conserved stoichiometry for homodimers and heterodimers; see Figure S1 in the Supplementary Material. The major difference between the results of the sequence‐based and structure‐based homology search methods is found in the negative heterodimers and monomers with ranking confidence above 0.8 (compare Figures 3e,f and S1e,f). This is logical, since we removed any monomers and heteromers that shared 90% or more *F‐ident* with a homomultimer during the dataset generation.

In summary, stoichiometry is poorly conserved in distant homologs for all types of proteins (monomers, heterodimers, and homodimers). At a TM‐score of 0.8, only about 60% of homo‐ and heterodimeric proteins have the same stoichiometry, whereas about 80% of monomers do. However, whether this reflects true changes in stoichiometry or uncertainty in PDB annotations needs further study. Further, it can be noted that for incorrectly classified proteins (both low‐scoring homodimers and high‐scoring monomers and heterodimers), the fraction of (close) homologs with “correct” stoichiometry is lower than for correctly classified proteins. This suggests we can use this information to improve classification, which is what we set out to do using the Logistic Regression described below.

### 3.4 Improved identification of homodimers

Next, we improve homodimer identification using logistic regression. First, we examined the performance of 70 features. In short, we used 24 features extracted from the interface, 27 homology stoichiometry‐based features, and 19 AlphaFold‐based features. Thereafter, we optimized feature selection and tuned the logistic regression model. Finally, we established a full prediction pipeline as described in Figure 4.

**FIGURE 4:**
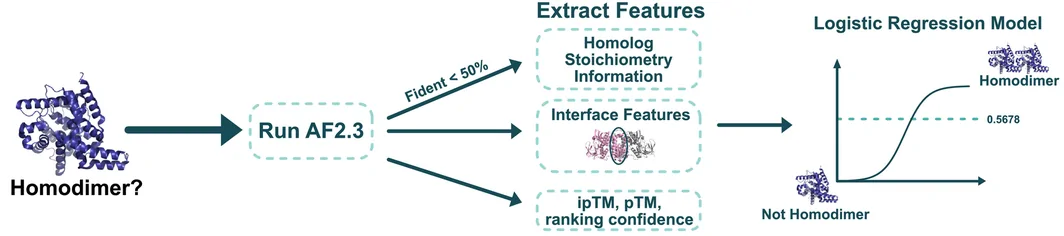
The protein of interest is first predicted as a homodimer using AlphaFold2.3. The input features for the logistic model consist of three parts. The first part is information about the stoichiometry of homologs extracted using Foldseek. Proteins with Fident above 0.5 are removed from the results so the model does not overfit to proteins with many homologs. The second feature group consists of information about the interfaces of the predicted models. Thirdly, AlphaFold's confidence metrics such as ranking confidence, ipTM, and pTM are used as input to the model. The final prediction of the model is whether the input sequence would form a homodimer.

#### 3.4.1 Performance of individual features

Several types of features, structural‐, AlphaFold‐ and homolog stoichiometry‐based, were used to improve the identification of homodimeric proteins; see Section 2 for details and Table 3 for a list. We tested each feature's ability to predict homodimers in the dataset, and the results are shown in Table S6. Many features correlate strongly, especially within the group containing AlphaFold confidence metrics and structural consensus. The AlphaFold‐based metrics had the highest AUPR, ranging from 0.78 to 0.80, with 0.80 the highest recorded. The ipSAE‐based features performed equally well, achieving 62% TPR at 5% FPR, with average ipSAE being the highest.

**TABLE 3:** Features included in the logistic regression models.

Feature	LogReg w H.S.I.^a^	LogReg w/o H.S.I.^a^
Homolog stoichiometry information		
HM fraction, TM‐score ≥0.2	✓	—
Multi fraction, TM‐score ≥0.0	✓	—
Multi fraction, TM‐score ≥0.1	✓	—
Multi fraction, TM‐score ≥0.3	✓	—
foldseek_specificity	✓	—
Interface features		
Best contact score max	✓	✓
Frac. buried polar (min)	—	✓
Total interaction area (max)	—	✓
AF confidence and structural consensus		
Average ipSAE	✓	—
Average pTM	—	✓
Average RC	—	✓
Max pTM	—	✓
Min ipTM	✓	✓
Min RC	✓	—
ipTM × RC product	✓	—
Structural consensus (max)	✓	✓
Structural consensus (mean)	✓	—
Structural consensus (min)	✓	—

*Note*: Both models are the ones with the best TPR@1%FPR on the test set.

^a^ Homolog stoichiometry information.

For the group of features based on homolog stoichiometry information, the higher TM‐score cut‐offs up to 0.8 seem to contain more valuable information, which is in line with Figure 3a,d. However, at TM‐score 0.9 the performance drops, which is most likely due to fewer matches being found at this cut‐off. The feature best TM‐score (all) seems to contain little information, since the AUPR is low (AUPR = 0.49) as shown in Table S6. However, it is still higher than a random classifier since, for AUPR (in contrast to area under the receiver–operator curve), a random classifier equals the prevalence of the positive class (in this case 0.344) (Saito & Rehmsmeier, 2015). This feature is just the score of the highest Foldseek hit regardless of its stoichiometry, which is understandably not very useful for homodimer prediction.

The interface features inspired by SPOC (except best pAE min) all have AUPR between 0.6 and 0.78, slightly below the performance of the AlphaFold confidence metrics. The features best pLDDT max and min contacts across predictions both achieve an AUPR of 0.78, which is very close to the highest value among the AlphaFold‐based metrics, 0.8. It is also notably higher than all homolog stoichiometry information‐based features.

Among the homolog stoichiometry‐ and AlphaFold‐derived features, the best‐performing feature is the product of the minimum ipTM and the ranking confidence ipTM rc prod. This feature achieves the highest Matthews correlation coefficient (MCC) and F1‐score but overall performs similarly to the minimum *ipTM* and *ranking confidence*, which is expected since the metric combines these two scores.

#### 3.4.2 Optimal TPR using logistic regression

We found the optimal feature combination using 6 feature‐selection tools from the scikit‐learn library (Pedregosa et al., 2011) on the training set with Stratified 5‐fold cross‐validation. The combinations that yielded the highest mean TPR at 1% and the highest mean AUPR across the five folds of the training set were selected. The performance is then reported on the test set in Table 4 and Figure 5. For more details on the feature selection methods explored, see Supplementary Material Section S3.2. Note that the numbers in Table 4 differ from those in Table 1 because Table 1 reports only test‐set performance.

**TABLE 4:** AUPR, F1, MCC, TPR@5%FPR and TPR@1%FPR on the held‐out test set.

Method	AUPR (↑)	F1 (↑)	MCC (↑)	TPR@5%FPR (↑)	TPR@1%FPR (↑)
RC (average)	0.80	0.40	0.40	0.61	0.26
RC (maximum)	0.80	0.36	0.37	0.60	0.23
RC (minimum)	0.80	0.42	0.41	0.62	0.27
ipTM (average)	0.81	0.40	0.40	0.62	0.26
ipTM (maximum)	0.80	0.37	0.38	0.60	0.23
ipTM (minimum)	0.80	0.42	0.41	0.63	0.27
ipSAE (average)	0.80	0.35	0.36	0.63	0.21
ipSAE (maximum)	0.80	0.35	0.36	0.61	0.22
ipSAE (minimum)	0.80	0.36	0.37	0.62	0.23
LR w/o homology (AUPR #1, *k* = 8)	0.81	0.41	0.41	0.63	0.26
LR w/o homology (TPR@1% #1, *k* = 3)	0.80	0.40	0.40	0.64	0.25
LR w/ homology (AUPR #1, *k* = 10)	**0.82**	0.48	0.46	0.65	0.32
LR w/ homology (TPR@1% #1, *k* = 13)	**0.82**	**0.52**	**0.49**	**0.66**	**0.36**

*Note*: Threshold determined at 1% FPR on the training set and applied to the test set.

**FIGURE 5:**
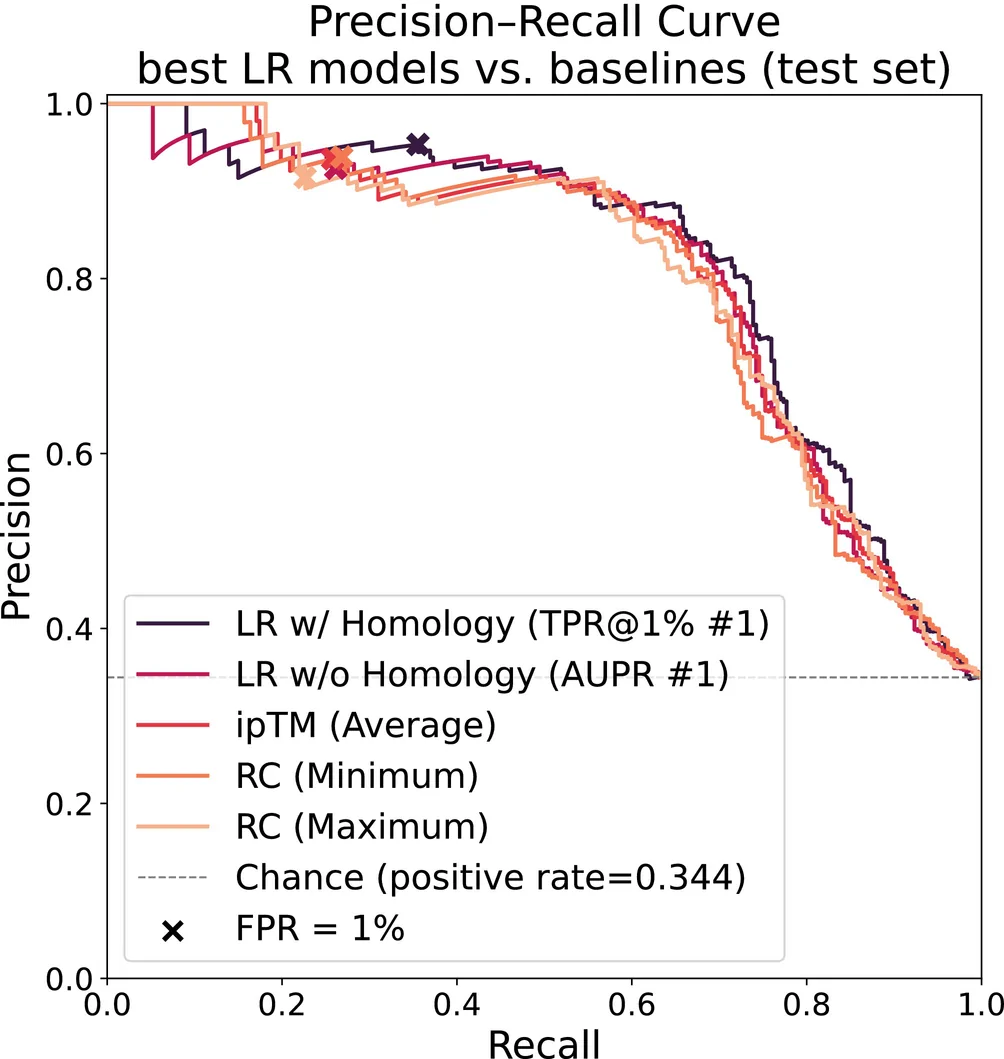
Precision‐recall curve for logistic regression (LR) models and default AlphaFold2.3 scores. The “X” mark is where the FPR is 1%.

Both models with homolog stoichiometry information improve homodimer prediction across all metrics, with the most notable improvement being for TPR@1%FPR, especially for the model based on this metric during cross‐validation. Using the McNemar statistical hypothesis test (Lachenbruch, 2014), the binary classification improvement at 1%FPR for the best homolog stoichiometry information‐based model (TPR@1% #1, *k* = 13) was significant with a *p*‐value of 0.0034. However, the difference in TPR@1%FPR itself was not statistically significant in a paired permutation test, and the corresponding bootstrap confidence interval included zero. The model without stoichiometry information among homologs did not show a statistically significant improvement over the best individual baseline in the continuous‐score comparisons.

Without homolog stoichiometry information, it seems difficult to improve homodimer prediction with this approach and the features collected in this study. An alternative approach would be to use a graph neural network or similar methods to analyze the interfaces of the predicted structures together with more advanced features such as ESM‐embeddings (Lin et al., 2023). This would also require an extension of the training dataset, possibly to include entries before the training cut‐off of AlphaFold.

Incorporating homolog stoichiometry information showed modest, non‐significant improvement. However, it should be noted that many similar structures were filtered out when employing a max *F‐ident* of 0.5 during both training and inference. Allowing higher sequence similarity between the query and similar structures during inference should further improve results.

## 4 CONCLUSIONS

In this study, we assessed AlphaFold's ability to identify proteins that form homodimers. First, we surprisingly found that AlphaFold produced a higher fraction of false positives for homodimers, that is, proteins that are either monomers or part of a heterodimeric protein pair, when predicted as homodimers. This is in stark contrast to the well‐studied ability of AlphaFold to identify heterodimeric interactions. We found that a possible reason is that many proteins with similar structures of these high‐scoring non‐homodimeric proteins form homomultimers (not only homodimers). This indicates that “stoichiometry” is not well conserved even among close homologs. Although some of these cases may reflect errors in PDB classifications, it is unlikely to be the full explanation.

Next, to reduce false positives, we incorporate stoichiometry information among homologs, interface, and AlphaFold‐based features into a logistic regression model and found that 13 features are sufficient for moderate homodimer prediction improvement. The biggest gain came from combining AlphaFold scores with homolog stoichiometry information; without the homolog stoichiometry information, no improvement over AlphaFold raw scores was seen. Using this information enables a logistic regression function to identify 36% of the homodimers at 1% FPR, up from 27%. However, because the TPR for heterodimers is 81%, there is still considerable room for improvement in the accurate identification of homodimers in future methods.

## AUTHOR CONTRIBUTIONS

**Sarah Narrowe:** Conceptualization; investigation; software; data curation; project administration; writing – review and editing; writing – original draft; methodology; visualization; validation; formal analysis. **Arne Elofsson:** Conceptualization; supervision; resources; funding acquisition; writing – review and editing.

## FUNDING INFORMATION

Vetenskapsrådet, grant no. 2021‐03979, the Knut and Alice Wallenberg Foundation, grant no. 2022.0032. SNIC, grant numbers NAISS2021/5‐297 and Berzelius‐2021‐29.

## CONFLICT OF INTEREST STATEMENT

The authors declare no conflicts of interest.

## Supporting information


**Figure S1.** In this figure, homologs were found on the sequence level using MMseqs2 instead of on the structural level. Instead of using TM‐score as a cutoff, the F‐ident metric was used. We can see a pattern similar to that of the structural homologs, with a few exceptions.
**Figure S2:** This is an extension of Figure 3 in the main text containing the distribution of all three stoichiometry classes, overall and below and above the threshold of 0.8 for the ranking confidence.
**Table S1:** Positive homodimers (n=716). PDB identifiers of the positive homodimers used to train and evaluate the classifier.
**Table S2:** Monomers (n=783). PDB identifiers of monomeric structures, used as negative examples for the homodimer classification task.
**Table S3:** Separated heterodimeric chains (n=584). PDB identifiers (with chain identifiers) of individual chains separated from heterodimeric complexes, used as negative examples for the homodimer classification task.
**Table S4:** Positive heterodimers (n=139). PDB identifiers of the positive heterodimers used to compare the performance of heterodimer and homodimer prediction of AlphaFold2.3.
**Table S5:** Negative heterodimers (n=293). Identifiers of shuffled (recombined) positive heterodimer pairs and monomers, used as negative examples for the heterodimer classification task.
**Table S6:** Results of individual feature performance collected for homodimer performance improvement.
**Table S7:** Explanation of each feature retrieved using code from SPOC. Taken from Table S3 of the supplementary material of Predictomes (Schmid & Walter, 2025).
**Table S8:** Results on the homodimeric dataset using different AlphaFold versions and QSProteome from Schweke et al. (2024). AlphaFold2.0 was run using ColabFold (Mirdita et al., 2022).
**Table S9:** Area under the precision‐recall curve (AUPR) recomputed at hypothetical signal‐to‐noise (positive:negative) ratios of 1:4, 1:10, 1:100, 1:1000. Precision is reweighted analytically from the empirical, ratio‐independent ROC curve. The best value for each row is marked in bold.
**Table S10:** Foldseek structural homologs at TM‐score ≥0.999 with MMseqs sequence identity (F‐ident), excluding homomultimer targets. One row per unique query–target PDB pair, with the best TM‐score kept. F‐ident is the maximum sequence identity found by MMseqs for that PDB pair; − indicates that the pair was not found in the MMseqs results.
**Table S11:** Filtering by SEQRES/ATOM length difference (≥5% threshold).
**Table S12:** Chain length filter (50–2000 aa).
**Table S13:** Cross‐dataset MMseqs2 filter (identity >90%).
**Table S14:** Heterodimer dataset after internal clustering.
**Table S15:** Heterodimer dataset after homomultimer filtering.
**Table S16:** Final dataset composition.

**Data S1.** Supporting Information.

**Data S2.** Supporting Information.

## Data Availability

All code and results are available on GitHub: https://github.com/SarahND97/alphafold-homodimers, and the predicted models, FASTA files, and MSAs are found on Zenodo, DOI: https://doi.org/10.5281/zenodo.22645239. URL: https://zenodo.org/records/22645239.

## References

[pro70873-bib-0001] Abramson J , Adler J , Dunger J , Evans R , Green T , Pritzel A , et al. Accurate structure prediction of biomolecular interactions with AlphaFold 3. Nature. 2024;630(8016):493–500. 10.1038/s41586-024-07487-w PMC11168924 38718835

[pro70873-bib-0002] Alberts B , Johnson A , Lewis J , Raff M , Roberts K , Walter P . Molecular biology of the cell. 4th ed., New York: Garland Science, 2002.

[pro70873-bib-0003] Anishchenko I , Ovchinnikov S , Kamisetty H , Baker D . Origins of coevolution between residues distant in protein 3D structures. Proc Natl Acad Sci. 2017;114(34):9122–9127. 10.1073/pnas.1702664114 PMC5576787 28784799

[pro70873-bib-0004] Bryant P , Pozzati G , Elofsson A . Improved prediction of protein‐protein interactions using AlphaFold2. Nat Commun. 2022;13(1):1265. 10.1038/s41467-022-28865-w PMC8913741 35273146

[pro70873-bib-0005] Cheng J , Novati G , Pan J , Bycroft C , Žemgulytė A , Applebaum T , et al. Accurate proteome‐wide missense variant effect prediction with AlphaMissense. Science. 2023;381(6664):eadg7492. 10.1126/science.adg7492 37733863

[pro70873-bib-0006] Dey S , Ritchie DW , Levy ED . PDB‐wide identification of biological assemblies from conserved quaternary structure geometry. Nat Methods. 2018;15(1):67–72. 10.1038/nmeth.4510 29155427

[pro70873-bib-0007] Dunbrack RL . Rēs ipSAE loquunt: what's wrong with AlphaFold's ipTM score and how to fix it. bioRxiv Preprint. 2025, 10.1101/2025.02.10.637595

[pro70873-bib-0008] Evans R , O'Neill M , Pritzel A , et al. Protein complex prediction with AlphaFold‐multimer. bioRxiv Preprint. 2021. https://www.biorxiv.org/content/10.1101/2021.10.04.463034v2

[pro70873-bib-0009] Fromm S , Ludaic M , Elofsson A . Evaluating deep learning based structure prediction methods on antibody‐antigen complexes. Bioinformatics. 2026;42(4):btag136. 10.1093/bioinformatics/btag136 PMC13061134 41863324

[pro70873-bib-0010] Jumper J , Evans R , Pritzel A , Green T , Figurnov M , Ronneberger O , et al. Highly accurate protein structure prediction with AlphaFold. Nature. 2021;596(7873):583–589. 10.1038/s41586-021-03819-2 PMC8371605 34265844

[pro70873-bib-0011] Kempen vM , Kim SS , Tumescheit C , et al. Fast and accurate protein structure search with Foldseek. Nat Biotechnol. 2023;42(2):243–246. 10.1038/s41587-023-01773-0 PMC10869269 37156916

[pro70873-bib-0012] Lachenbruch PA . McNemar test. Chichester, UK: John Wiley & Sons, Ltd; 2014.

[pro70873-bib-0013] Lin Z , Akin H , Rao R , Hie B , Zhu Z , Lu W , et al. Evolutionary‐scale prediction of atomic‐level protein structure with a language model. Science. 2023;379(6637):1123–1130. 10.1126/science.ade2574 36927031

[pro70873-bib-0014] Martin J . AlphaFold2 predicts whether proteins interact amidst confounding structural compatibility. J Chem Inf Model. 2024;64(5):1473–1480. 10.1021/acs.jcim.3c01805 38373070

[pro70873-bib-0015] Mirdita M , Schütze K , Moriwaki Y , Heo L , Ovchinnikov S , Steinegger M . ColabFold: making protein folding accessible to all. Nat Methods. 2022;19(6):679–682. 10.1038/s41592-022-01488-1 PMC9184281 35637307

[pro70873-bib-0016] Mitternacht S . FreeSASA: an open source C library for solvent accessible surface area calculations. F1000Res. 2016;5:189. 10.12688/f1000research.7931.1 PMC4776673 26973785

[pro70873-bib-0017] Pedregosa F , Varoquaux G , Gramfort A , et al. Scikit‐learn: machine learning in python. J Mach Learn Res. 2011;12:2825–2830.

[pro70873-bib-0018] Pozzati G , Zhu W , Bassot C , Lamb J , Kundrotas P , Elofsson A . Limits and potential of combined folding and docking. Bioinformatics. 2022;38:954–961. 10.1093/bioinformatics/btab760 PMC8796369 34788800

[pro70873-bib-0019] Saito T , Rehmsmeier M . The precision‐recall plot is more informative than the ROC plot when evaluating binary classifiers on imbalanced datasets. PLoS One. 2015;10(3):e0118432. 10.1371/journal.pone.0118432 PMC4349800 25738806

[pro70873-bib-0020] Schmid EW , Walter JC . Predictomes, a classifier‐curated database of AlphaFold‐modeled protein‐protein interactions. Mol Cell. 2025;85(6):1216–1232.e5. 10.1016/j.molcel.2025.01.034 PMC11931459 40015271

[pro70873-bib-0021] Schweke H , Pacesa M , Levin T , Goverde CA , Kumar P , Duhoo Y , et al. An atlas of protein homo‐oligomerization across domains of life. Cell. 2024;187(4):999–1010. 10.1016/j.cell.2024.01.022 38325366

[pro70873-bib-0022] Steinegger M , Söding J . MMseqs2 enables sensitive protein sequence searching for the analysis of massive data sets. Nat Biotechnol. 2017;35(11):1026–1028. 10.1038/nbt.3988 29035372

[pro70873-bib-0023] Szklarczyk D , Kirsch R , Koutrouli M , Nastou K , Mehryary F , Hachilif R , et al. The STRING database in 2023: protein–protein association networks and functional enrichment analyses for any sequenced genome of interest. Nucleic Acids Res. 2023;51(D1):D638–D646. 10.1093/nar/gkac1000 PMC9825434 36370105

[pro70873-bib-0024] Westbrook J , Feng Z , Jain S , Bhat TN , Thanki N , Ravichandran V , et al. The Protein Data Bank: unifying the archive. Nucleic Acids Res. 2002;30(1):245–248. 10.1093/nar/30.1.245 PMC99110 11752306

[pro70873-bib-0025] Xu Q , Dunbrack J . The protein common interface database (ProtCID)—a comprehensive database of interactions of homologous proteins in multiple crystal forms. Nucleic Acids Res. 2011;39(suppl_1):D761–D770. 10.1093/nar/gkq1059 PMC3013667 21036862

[pro70873-bib-0026] Zhang C , Shine M , Pyle AM , Zhang Y . US‐align: universal structure alignments of proteins, nucleic acids, and macromolecular complexes. Nat Methods. 2022;19(9):1109–1115. 10.1038/s41592-022-01585-1 36038728

[pro70873-bib-0027] Zhang J , Humphreys IR , Pei J , Kim J , Choi C , Yuan R , et al. Predicting protein‐protein interactions in the human proteome. Science. 2025;390(6771):eadt1630. 10.1126/science.adt1630 PMC13281779 40997207

