# Supplementary material for: Reliable identification of homodimers using AlphaFold
Source: Protein Sci. 2026 Nov 10;35(12):e70873. doi: 10.1002/pro.70873 (PMC13749628; doi:10.1002/pro.70873)
Supplement: Supplementary file 1 [file PRO-35-e70873-s001.pdf]

S:1 | SUPPLEMENTARY FIGURES

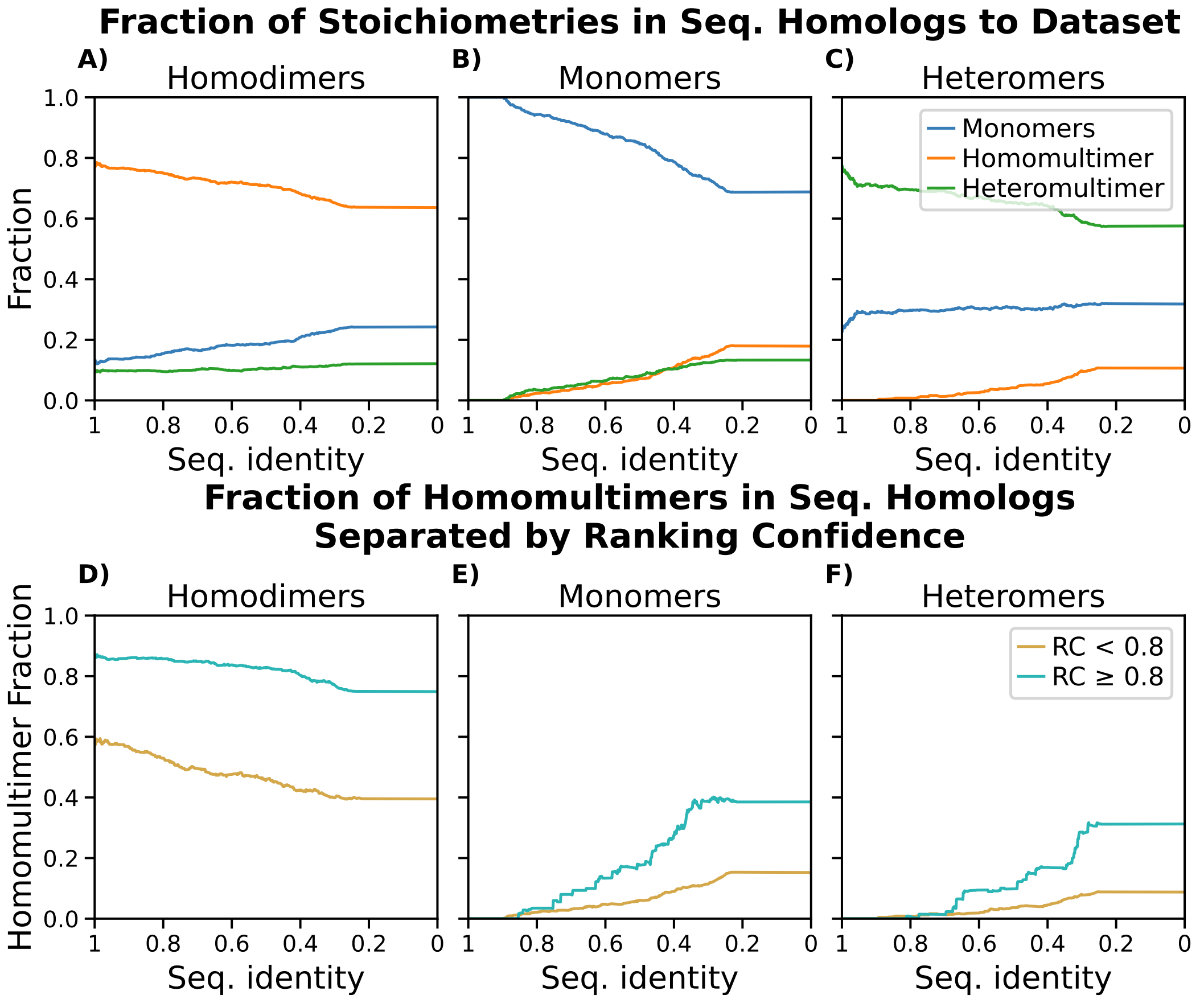

FIGURE S1 In this figure, homologs were found on the sequence level using MMseqs2 instead of on the structural level. Instead of using TM-score as a cutoff, the F-ident metric was used. We can see a pattern similar to that of the structural homologs, with a few exceptions.

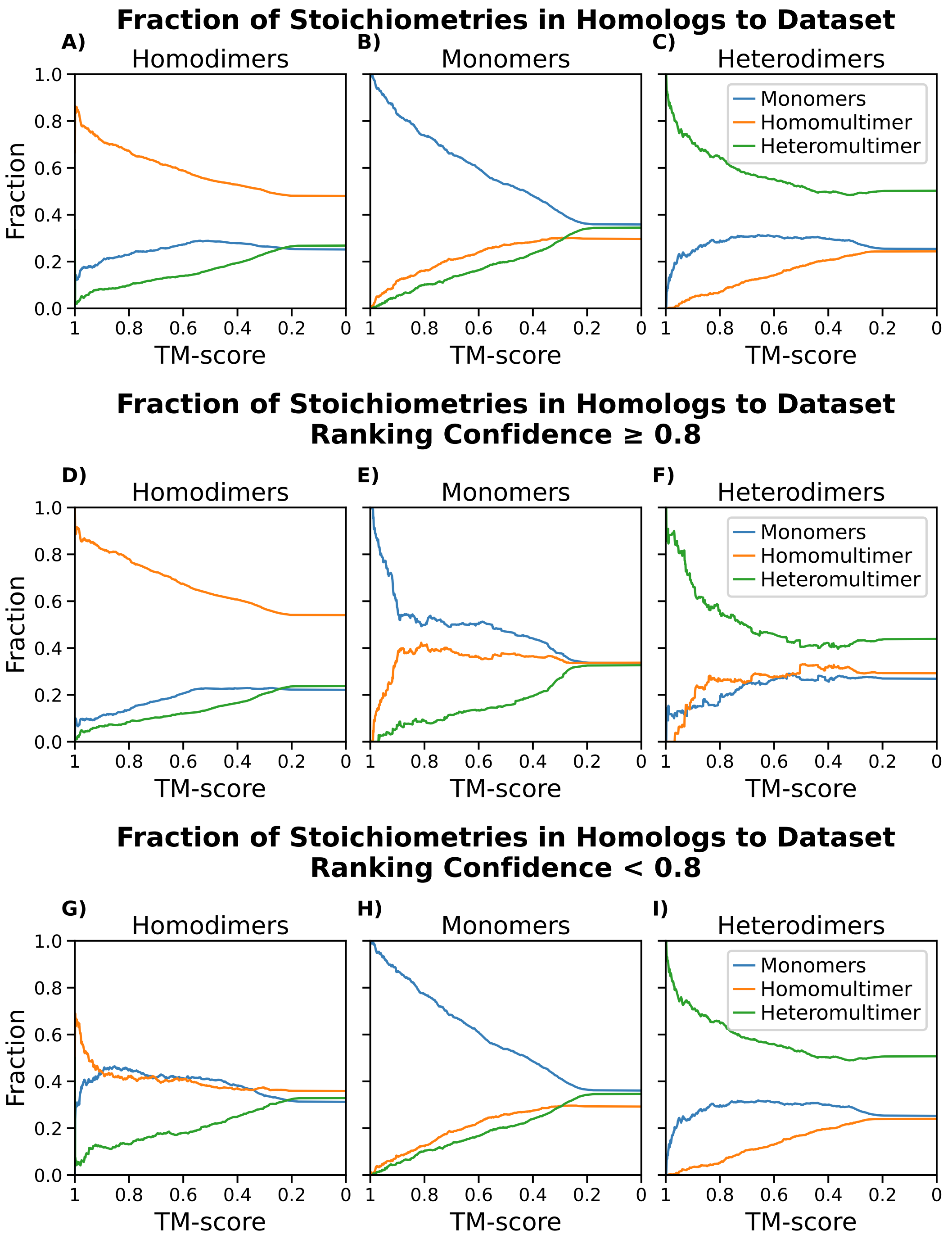

FIGURE S2 This is an extension of Figure 3 in the main text containing the distribution of all three stoichiometry classes, overall and below and above the threshold of 0.8 for the ranking confidence.

S:2 | SUPPLEMENTARY TABLES

Positive homodimers

10jx, 10xb, 10xe, 10yv, 11bd, 12kh, 13bb, 13jb, 21ih, 22fg, 23fh, 28pb, 6z03, 7aal, 7aik, 7arz, 7awv, 7azq, 7b1d, 7b4q, 7b7t, 7b9p, 7ban, 7bih, 7d50, 7d54, 7da9, 7dck, 7dfe, 7dkb, 7dnt, 7dut, 7e00, 7e3q, 7e4n, 7e4v, 7e52, 7e5m, 7e8j, 7e8r, 7ead, 7ebo, 7ed2, 7eg5, 7elt, 7epq, 7erl, 7erq, 7erv, 7ets, 7ety, 7eyp, 7ezy, 7f0h, 7f47, 7f6w, 7f7p, 7f84, 7fco, 7fct, 7fh3, 7fib, 7fj7, 7hk6, 7l75, 7l8n, 7lat, 7ltw, 7ly6, 7m3h, 7mjc, 7mo6, 7ms2, 7msn, 7mu0, 7mzy, 7n79, 7n7g, 7ncy, 7nto, 7o6w, 7oc3, 7ojr, 7ovp, 7oyl, 7p9k, 7pcn, 7pcr, 7pgf, 7poe, 7pow, 7pps, 7pq3, 7pug, 7pwe, 7pzo, 7q0q, 7q4h, 7q6a, 7qa1, 7qcn, 7qd1, 7qoc, 7qri, 7qul, 7qxo, 7r09, 7r3i, 7r6g, 7r9b, 7rhw, 7rmx, 7rwk, 7s5i, 7s6e, 7s8k, 7spn, 7spq, 7stt, 7t2f, 7t63, 7t7j, 7t88, 7t8i, 7t8l, 7t8o, 7tak, 7tcm, 7tdq, 7tl5, 7tmg, 7tot, 7trw, 7tvm, 7u2r, 7uaw, 7uln, 7unh, 7unj, 7uoc, 7ux7, 7v3d, 7v4o, 7v4s, 7v58, 7v8h, 7va2, 7vaa, 7vcp, 7vem, 7vf6, 7vid, 7vkw, 7vm0, 7vqk, 7vr2, 7vs6, 7vua, 7vwk, 7vwt, 7vyj, 7vzo, 7w5q, 7w70, 7w93, 7w9x, 7wbc, 7wbt, 7wf9, 7wgu, 7wn9, 7wuy, 7wvz, 7ww2, 7wwt, 7wze, 7x0q, 7x53, 7x7i, 7x7w, 7x80, 7xao, 7xc4, 7xdu, 7xfs, 7xfu, 7xgm, 7xi6, 7xll, 7xmw, 7xn2, 7xpi, 7xpo, 7xrb, 7xro, 7xuj, 7xux, 7xv2, 7xv9, 7xxx, 7xzp, 7y3p, 7y3w, 7y4o, 7y6o, 7y73, 7y7u, 7y9a, 7ybd, 7yeo, 7yh2, 7yic, 7yii, 7yji, 7ykc, 7yq0, 7yqr, 7yr9, 7ysa, 7yuj, 7ywq, 7yxk, 7z5p, 7z9a, 7zc8, 7zcv, 7ze0, 7zhu, 7zkh, 7zm9, 7znm, 7zs3, 8a2g, 8a30, 8a6v, 8a8d, 8a90, 8a9n, 8aep, 8aew, 8ahr, 8amy, 8avh, 8ayv, 8b0v, 8b1n, 8b1v, 8b5v, 8b63, 8b73, 8bbq, 8bbu, 8bcs, 8beg, 8bic, 8bit, 8bj2, 8bnb, 8bp5, 8byh, 8c46, 8c4l, 8c79, 8c9s, 8chw, 8cid, 8ciw, 8ckh, 8clz, 8cmx, 8cok, 8cqs, 8cqv, 8crh, 8crv, 8cxl, 8d27, 8d4w, 8d8s, 8dci, 8deb, 8dek, 8dh7, 8dkd, 8dkp, 8dlc, 8dos, 8dqb, 8dqq, 8du5, 8ecp, 8eei, 8egz, 8epz, 8er5, 8eun, 8evm, 8f5m, 8f8o, 8few, 8fk2, 8ftr, 8fvt, 8fvx, 8fx9, 8g1h, 8g48, 8g9k, 8gad, 8gpv, 8gy0, 8gy8, 8gyg, 8gyi, 8gyw, 8h0r, 8h1n, 8h92, 8har, 8haw, 8he6, 8hgv, 8hjw, 8hm5, 8hn2, 8ho2, 8hpb, 8htw, 8hvq, 8hxs, 8hy9, 8i6i, 8i80, 8i9i, 8ic6, 8ieu, 8ilj, 8ill, 8ioa, 8ioi, 8is6, 8ito, 8iw2, 8iwq, 8ix7, 8iyi, 8j2w, 8j59, 8j5f, 8jd7, 8je4, 8je7, 8jhh, 8ji1, 8jnx, 8jqe, 8jsk, 8jtu, 8ju7, 8jus, 8jyb, 8jyv, 8k03, 8k1b, 8k3b, 8k40, 8k55, 8k74, 8k76, 8k7y, 8k8g, 8ken, 8khg, 8oqx, 8p0s, 8p6k, 8pb8, 8pek, 8png, 8pnx, 8pqp, 8puj, 8puz, 8pxf, 8pz3, 8q1o, 8q28, 8q3y, 8q50, 8q9v, 8qbf, 8qcj, 8qdq, 8qf0, 8qf3, 8qij, 8qkw, 8qlh, 8qll, 8qmf, 8qpl, 8qps, 8qpt, 8qqh, 8qyg, 8qzy, 8r3n, 8r7i, 8ray, 8rb7, 8rdm, 8rg5, 8rqy, 8rtv, 8s0u, 8s1j, 8s2f, 8s9w, 8scd, 8sky, 8snz, 8srt, 8sxy, 8t7m, 8ta2, 8ted, 8tfz, 8tka, 8tlu, 8tn8, 8twq, 8txl, 8u0g, 8u0z, 8u99, 8udb, 8un2, 8upb, 8upk, 8urt, 8utk, 8uub, 8uyr, 8v14, 8v4g, 8va3, 8vd9, 8vpl, 8vrx, 8vva, 8vwr, 8w7y, 8wbb, 8wbr, 8wc0, 8wdq, 8weu, 8wfc, 8wfo, 8wjb, 8wkj, 8wkx, 8wl1, 8wmy, 8woj, 8wp6, 8wqp, 8wrf, 8wwg, 8x6k, 8x6v, 8xdr, 8xf1, 8xh6, 8xha, 8xjb, 8xsh, 8xtb, 8xtf, 8xtv, 8xuo, 8y1x, 8y8m, 8y9q, 8yfd, 8yk5, 8ymk, 8yqj, 8yuf, 8yv5, 8yye, 8yzg, 8z28, 8z2m, 8zb2, 8zc8, 8zec, 8zgb, 8zgi, 8zlc, 8zn0, 8zoo, 8zpi, 8zr6, 9ard, 9au3, 9bce, 9bd4, 9be2, 9bgp, 9biw, 9br6, 9c8w, 9c9a, 9cd2, 9cgo, 9cp8, 9cv6, 9cx8, 9cxr, 9ddl, 9dnk, 9dq0, 9dqm, 9dr0, 9dt4, 9e4s, 9e72, 9e92, 9ea4, 9edq, 9efa, 9em9, 9eoi, 9eqo, 9erh, 9etw, 9eu1, 9ewr, 9ezx, 9f1t, 9f7e, 9fdo, 9ffk, 9fiz, 9fj8, 9fmn, 9fov, 9fp4, 9frr, 9fsg, 9fzn, 9g04, 9g0k, 9g92, 9g9u, 9g9v, 9gcj, 9ggo, 9gh9, 9gol, 9gqr, 9grw, 9gtj, 9gun, 9gzg, 9h3a, 9h8u, 9he0, 9hmy, 9hqi, 9hr1, 9hrs, 9ht6, 9htw, 9hvh, 9i0g, 9i1x, 9ilw, 9imj, 9imr, 9ioq, 9iqc, 9iqh, 9ir0, 9ir6, 9ire, 9irq, 9iux, 9ivz, 9j0t, 9j2e, 9j5q, 9j7w, 9jc7, 9jca, 9jfb, 9jfl, 9jgb, 9jjv, 9jnr, 9jq8, 9jqs, 9jrr, 9jzo, 9k05, 9k35, 9kax, 9kbc, 9kbq, 9kbt, 9ke2, 9kk8, 9kkt, 9kls, 9knp, 9kns, 9kpq, 9kq4, 9krc, 9kuj, 9kva, 9kw9, 9kyt, 9l1i, 9l75, 9lgt, 9ll4, 9ll6, 9lth, 9lyy, 9m0b, 9m2i, 9m7e, 9m8t, 9ma8, 9mcd, 9mgt, 9mpy, 9n03, 9n2k, 9n2w, 9n3v, 9n59, 9na0, 9nli, 9nt5, 9nxl, 9nxw, 9nyu, 9o90, 9of1, 9oqs, 9ozv, 9p9p, 9phg, 9pqf, 9pv2, 9q35, 9qd0, 9qd1, 9qdw, 9qih, 9qko, 9qof, 9qqk, 9qr2, 9qrf, 9qup, 9r8s, 9rfy, 9rpl, 9rui, 9rxh, 9s7k, 9si6, 9sqs, 9sxb, 9t62, 9ueq, 9uf7, 9ufq, 9uhd, 9uib, 9uu2, 9uu7, 9uu9, 9uz6, 9v0w, 9v60, 9vbg, 9vbm, 9vcd, 9vg8, 9vhm, 9vi5, 9vkn, 9vlf, 9voa, 9vp8, 9vvt, 9vzo, 9w60, 9wat, 9wh7, 9wq2, 9wt2, 9wuc, 9x4v, 9xgm, 9xzp, 9y43, 9z71, 9zdi, 9zmy, 9zo1.

TABLE S1 Positive homodimers ($n = 716$). PDB identifiers of the positive homodimers used to train and evaluate the classifier.

Monomers

10pa, 10ps, 10yy, 11ch, 11gz, 21xq, 21ze, 22gu, 22oc, 23ri, 24aj, 24nk, 24su, 25wn, 26jk, 27af, 28oj, 28wn, 29iv, 29sb, 6wqb, 6x6n, 6yrr, 6zbs, 6zuz, 7aaf, 7aao, 7ac1, 7b29, 7b3a, 7b76, 7bb9, 7bcj, 7d6l, 7d8k, 7dab, 7dcn, 7dht, 7djx, 7dmf, 7dms, 7e2b, 7e42, 7e5j, 7e8l, 7eau, 7eb1, 7ecd, 7ecq, 7ee2, 7eet, 7ejg, 7eo3, 7es4, 7evh, 7exo, 7exx, 7eyo, 7f2g, 7f6r, 7f7n, 7fa1, 7fac, 7fav, 7fc7, 7fcc, 7fev, 7fgz, 7fh7, 7fi0, 7fia, 7ko9, 7kob, 7kom, 7kuw, 7kw9, 7kyd, 7l6j, 7ldf, 7lns, 7lxk, 7m5t, 7mcc, 7mkr, 7mn2, 7mqq, 7mu9, 7mxk, 7n11, 7n45, 7n99, 7nde, 7nek, 7nf9, 7nqe, 7nqg, 7ny0, 7nzc, 7nzd, 7o0b, 7o4n, 7obm, 7ofn, 7oio, 7olb, 7omk, 7ouc, 7p1q, 7p20, 7p3u, 7p4n, 7pb9, 7pbh, 7pc1, 7pk0, 7pl2, 7pmp, 7pqi, 7pqw, 7prd, 7pre, 7puj, 7pup, 7pvc, 7pvr, 7pw1, 7q1w, 7q58, 7q6g, 7q71, 7qa5, 7qab, 7qb0, 7qb8, 7qcc, 7qei, 7qil, 7qp3, 7qpl, 7qrw, 7qs2, 7qsu, 7qu0, 7qvd, 7qyi, 7r25, 7r3m, 7r6p, 7r8k, 7rb4, 7red, 7riz, 7rkb, 7rmy, 7rom, 7ry6, 7rz3, 7rzm, 7s5l, 7s6f, 7s9y, 7sew, 7siq, 7sxb, 7sxi, 7t03, 7t1m, 7t27, 7t5t, 7t5u, 7t7z, 7tbr, 7te2, 7tfq, 7tj3, 7tjb, 7tlx, 7tm4, 7tog, 7tpu, 7ty1, 7u0v, 7u1y, 7u37, 7ugc, 7uk3, 7uk7, 7ukb, 7uo6, 7upv, 7ur8, 7urp, 7uv1, 7uv2, 7uwv, 7uwy, 7uwz, 7v40, 7v6m, 7v8w, 7vc7, 7vep, 7ver, 7vfc, 7vfr, 7vgc, 7vgm, 7vkv, 7vlm, 7vnx, 7vqv, 7vs2, 7vtk, 7vty, 7vu3, 7vzm, 7w05, 7w1l, 7w3w, 7w86, 7w8f, 7wa9, 7wab, 7wao, 7waw, 7wbo, 7whm, 7wkf, 7wkh, 7wmy, 7wrn, 7wvr, 7wwc, 7wz5, 7x15, 7x4g, 7xc0, 7xcd, 7xhz, 7xks, 7xmh, 7xqz, 7xrj, 7xrw, 7xx8, 7y1u, 7y47, 7y4h, 7y4n, 7y54, 7y5r, 7y7n, 7yd4, 7ygk, 7yhr, 7yib, 7yjl, 7ylr, 7ymb, 7ymo, 7ypl, 7yzs, 7z3c, 7z3s, 7z65, 7z66, 7zc2, 7zck, 7zgm, 7zhe, 7zjb, 7zjy, 7zk0, 7zkd, 7znp, 7znz, 7zok, 7zpt, 7ztf, 7zya, 8a06, 8a0h, 8af1, 8agf, 8ah9, 8ajp, 8akp, 8alu, 8ap5, 8ar0, 8ar3, 8ate, 8axj, 8axy, 8azb, 8b2e, 8b3y, 8b4u, 8b7t, 8b97, 8bau, 8bdp, 8bgk, 8bjo, 8bpz, 8bqa, 8bvi, 8bvo, 8bvp, 8bw6, 8bwb, 8bxr, 8bxu, 8byk, 8bzq, 8c18, 8c4y, 8c7d, 8c7k, 8c8v, 8ch3, 8ckn, 8cnr, 8cnt, 8cqh, 8crj, 8cx6, 8d00, 8d6m, 8dc1, 8di1, 8dj8, 8djt, 8dld, 8dmf, 8dp2, 8dqd, 8drg, 8dv0, 8dwq, 8e18, 8e22, 8e4v, 8e5i, 8e7f, 8et6, 8ew9, 8f5d, 8f9o, 8f9y, 8fa3, 8fds, 8feb, 8fnx, 8fxu, 8fz8, 8g1m, 8g22, 8g64, 8g6p, 8ga7, 8gac, 8gbe, 8gc6, 8gf3, 8gjw, 8gqq, 8gs7, 8gsa, 8gtz, 8guu, 8gxd, 8gxt, 8gyn, 8h2f, 8h3j, 8h4m, 8h6r, 8hea, 8hkb, 8hnq, 8hnr, 8hok, 8ht3, 8hvb, 8hw0, 8i0a, 8i0d, 8i25, 8i53, 8id0, 8ii8, 8ikx, 8ilz, 8imq, 8iwd, 8j3n, 8j3q, 8j3v, 8j4w, 8j6e, 8ja6, 8jbr, 8jcq, 8jdc, 8jh8, 8jn0, 8jnt, 8jpv, 8jrm, 8jst, 8ju8, 8jv2, 8jvc, 8k2f, 8k2j, 8k2m, 8k5q, 8k7o, 8k7z, 8k8i, 8kb2, 8kh1, 8ose, 8oug, 8ouk, 8ov0, 8oys, 8ozz, 8p2a, 8p3b, 8p66, 8p7g, 8p8e, 8pbv, 8pof, 8pui, 8pvw, 8pyy, 8q5b, 8q8i, 8qhh, 8qjl, 8qkd, 8qnj, 8qnv, 8qpd, 8qq7, 8qrx, 8qsx, 8qve, 8qwt, 8qxi, 8qzu, 8r2c, 8r6b, 8r6n, 8r73, 8r8a, 8ra0, 8rd6, 8re2, 8rp9, 8ryz, 8rzf, 8s6c, 8s89, 8slv, 8sqx, 8suc, 8t2j, 8t41, 8t7w, 8t8l, 8tb1, 8tbx, 8tg0, 8tjg, 8tse, 8tw1, 8twg, 8tzm, 8u01, 8u0i, 8u1i, 8u49, 8uag, 8ubh, 8ufe, 8upi, 8us3, 8us4, 8uvv, 8v4w, 8v9u, 8vby, 8vc8, 8vcw, 8vdk, 8vez, 8vni, 8vrc, 8vuk, 8w98, 8wcf, 8wjh, 8wkc, 8wkh, 8wni, 8wom, 8wrx, 8wth, 8wtl, 8wzl, 8wzy, 8x1a, 8x1b, 8x1i, 8x2v, 8x3e, 8x4k, 8x67, 8x74, 8xcl, 8xe8, 8xgz, 8xhx, 8xik, 8xiv, 8xjg, 8xm1, 8xtt, 8xuk, 8xuw, 8xvo, 8y33, 8y4l, 8y4z, 8yg0, 8yk7, 8yke, 8yoa, 8yt7, 8yzn, 8z0n, 8z4q, 8zeq, 8zic, 8zna, 8znu, 8zop, 8zu1, 8zvc, 8zxg, 9atx, 9av6, 9avh, 9avk, 9az1, 9b1v, 9b3n, 9b53, 9b99, 9bal, 9bi7, 9biz, 9bug, 9byu, 9bzb, 9bzr, 9c0n, 9c14, 9c3d, 9c9n, 9ce9, 9cfj, 9cg1, 9cg2, 9cic, 9cj0, 9d01, 9d1j, 9d8a, 9d91, 9dcn, 9dea, 9ded, 9dee, 9dev, 9dm3, 9dsy, 9dtk, 9dug, 9e32, 9e9n, 9ef8, 9ehu, 9eid, 9emo, 9etk, 9exk, 9f4c, 9fcf, 9fdg, 9fej, 9ffe, 9fho, 9fjl, 9flg, 9fo1, 9fp9, 9frc, 9frq, 9fsj, 9ftd, 9fu8, 9fw5, 9fw7, 9fwa, 9fxq, 9fym, 9fzm, 9g7n, 9gar, 9gce, 9gl1, 9gly, 9gmj, 9gr8, 9gz5, 9h63, 9h8z, 9h9b, 9hb2, 9hho, 9hhu, 9hk0, 9hkj, 9hl5, 9hmb, 9hmo, 9hnj, 9hnn, 9hsa, 9hta, 9htu, 9hw9, 9i38, 9i44, 9iba, 9ibo, 9ic4, 9ifr, 9iuh, 9iuw, 9iy9, 9iz8, 9j0u, 9j7j, 9j7v, 9jak, 9jb3, 9jkp, 9jl3, 9jlv, 9jql, 9jtq, 9jvc, 9jwt, 9jx4, 9k3o, 9k97, 9kb3, 9kcs, 9kf2, 9kf4, 9kfy, 9kgz, 9kn2, 9ksm, 9kvn, 9kvu, 9kz6, 9l1v, 9l7l, 9l8t, 9lb6, 9lhs, 9lhu, 9lif, 9lnf, 9lni, 9lpb, 9lpt, 9lqb, 9ls6, 9ls8, 9lt9, 9luz, 9lvn, 9lx1, 9m0m, 9m27, 9m9g, 9mg4, 9mrc, 9mug, 9n1c, 9n1s, 9n1u, 9n3f, 9n8r, 9nbw, 9nia, 9o00, 9o3q, 9o5f, 9o99, 9oji, 9omb, 9p6w, 9pgb, 9pyv, 9q2g, 9q5u, 9qdh, 9qdt, 9qrg, 9qs1, 9quz, 9qvb, 9qye, 9r53, 9r8p, 9rb0, 9rs4, 9rxe, 9rxr, 9s11, 9s6a, 9s8y, 9sd5, 9slr, 9sn1, 9szu, 9t0n, 9u9n, 9ub0, 9ubb, 9udh, 9ujo, 9ukw, 9ul7, 9uml, 9uo7, 9ups, 9us5, 9uv0, 9v0m, 9v3a, 9v4l, 9vdl, 9vex, 9vsj, 9vyi, 9w4x, 9wjk, 9wsc, 9x1x, 9x43, 9x81, 9x8f, 9xpd, 9y6i, 9yc2, 9yd7, 9yv5, 9z30, 9zdo, 9zk5, 9zmu, 9zn4, 9zsh.

TABLE S2 Monomers ($n = 783$). PDB identifiers of monomeric structures, used as negative examples for the homodimer classification task.

Separated heterodimeric chains

10vb_A, 24rc_B, 6zh1_A, 7a67_A, 7a67_B, 7acw_A, 7acw_B, 7b2t_A, 7dn8_A, 7dnm_A, 7dnm_B, 7drb_A, 7drb_B, 7e1s_A, 7e5z_A, 7e5z_B, 7ebk_A, 7egt_B, 7eqx_A, 7eqx_B, 7et0_A, 7et0_B, 7ev8_A, 7f0f_A, 7f28_A, 7f28_B, 7f4t_B, 7f5k_A, 7f87_A, 7fax_A, 7fi3_A, 7g83_B, 7mkk_A, 7mni_A, 7mny_B, 7mon_B, 7mvu_B, 7mw1_B, 7mwq_A, 7n0e_B, 7n3t_B, 7n88_A, 7oje_A, 7oju_A, 7opb_B, 7opo_B, 7ox5_A, 7ox5_B, 7p0l_A, 7pdy_B, 7pk4_B, 7pmx_A, 7pul_A, 7py4_A, 7q42_A, 7q72_A, 7q72_B, 7qdw_A, 7qm2_B, 7qpi_A, 7qqy_A, 7quu_A, 7qv8_A, 7r49_A, 7r49_B, 7r86_B, 7rae_A, 7rb3_A, 7rb3_B, 7rc6_A, 7rt7_A, 7rt7_B, 7rup_A, 7rxq_A, 7s4n_A, 7s9v_A, 7s9v_B, 7sjq_B, 7spp_B, 7sxg_A, 7t6a_A, 7tcr_A, 7tcr_B, 7thn_A, 7tj4_A, 7tj4_B, 7tyd_B, 7tzh_B, 7u5o_A, 7u61_A, 7udj_B, 7udk_A, 7ue2_A, 7ui8_A, 7um4_A, 7uma_A, 7uve_A, 7v6b_B, 7vbq_B, 7vcr_B, 7vd7_A, 7vg7_B, 7vgh_A, 7vmx_A, 7vnb_A, 7vni_A, 7vsi_A, 7vsi_B, 7vyw_A, 7w3u_A, 7w4z_B, 7w5m_A, 7wdk_A, 7weg_A, 7wfc_A, 7whf_B, 7wrs_A, 7wrs_B, 7wul_B, 7wwq_A, 7x5c_A, 7x9s_A, 7xad_B, 7xae_B, 7xdd_A, 7xge_A, 7xky_A, 7xoi_B, 7xpc_A, 7xpc_B, 7xpk_A, 7xvk_A, 7xyq_B, 7y6b_A, 7y6b_B, 7y8u_A, 7y8u_B, 7y9c_A, 7ycj_B, 7yh8_A, 7yk3_A, 7yk3_B, 7ykg_A, 7yls_A, 7yls_B, 7yo3_B, 7yta_A, 7ytt_A, 7ytt_B, 7yx8_A, 7zb1_A, 7zch_A, 7zhm_A, 7zhm_B, 7zhr_B, 7zjv_A, 7zkw_A, 7zsc_A, 7zsd_B, 7zvc_A, 8a05_A, 8a4a_A, 8a60_B, 8a82_A, 8a82_B, 8abq_B, 8ack_A, 8ad9_A, 8adb_A, 8agy_A, 8aim_A, 8ain_B, 8ako_B, 8alk_A, 8alo_B, 8alz_B, 8arc_A, 8arc_B, 8arn_A, 8b0u_A, 8b0u_B, 8b14_B, 8b2f_A, 8b2q_B, 8b4o_A, 8b4o_B, 8ba1_A, 8ba1_B, 8bav_B, 8bbj_B, 8bfj_A, 8bgm_A, 8bgm_B, 8blw_A, 8blw_B, 8boz_A, 8boz_B, 8br1_B, 8brh_A, 8bs9_A, 8bv0_B, 8bv7_A, 8bvr_A, 8byp_A, 8c12_B, 8c8g_B, 8cem_A, 8cem_B, 8cjb_A, 8cm0_A, 8ct8_A, 8ct8_B, 8cws_B, 8cxj_B, 8cz9_A, 8d4s_B, 8ds8_A, 8dye_B, 8e51_A, 8e51_B, 8ehe_A, 8ehe_B, 8eho_A, 8enb_A, 8enb_B, 8exf_A, 8ey4_A, 8ey4_B, 8ezs_B, 8f6d_B, 8fex_A, 8fex_B, 8fg6_B, 8fn9_A, 8fn9_B, 8fss_A, 8g0k_A, 8g0k_B, 8g0p_A, 8g0p_B, 8g4k_A, 8gab_A, 8gja_A, 8gja_B, 8gjg_A, 8gji_A, 8gkq_A, 8gkq_B, 8glc_A, 8gm8_A, 8gp6_A, 8gp6_B, 8gqe_A, 8gs1_A, 8gs1_B, 8h0n_A, 8hci_A, 8hci_B, 8hf3_A, 8hf3_B, 8hfp_A, 8hfp_B, 8hm3_A, 8hm3_B, 8hsb_A, 8hsb_B, 8htd_A, 8i2f_B, 8i3g_A, 8i9q_B, 8ij9_B, 8ipl_B, 8ipr_A, 8ipr_B, 8iqs_B, 8ir4_A, 8j1i_B, 8j5u_A, 8j64_B, 8jc5_B, 8jca_B, 8jfw_B, 8jg4_A, 8jkz_A, 8jkz_B, 8jmr_A, 8jo4_A, 8jsu_B, 8jzd_A, 8k0e_A, 8k0h_A, 8k0h_B, 8k1l_A, 8k1l_B, 8k4r_A, 8odp_A, 8ogi_A, 8ogi_B, 8oi3_B, 8onu_A, 8onw_A, 8onw_B, 8or7_A, 8or7_B, 8orn_A, 8orn_B, 8os1_A, 8ows_A, 8ows_B, 8p0q_A, 8pfd_B, 8pq7_A, 8pq7_B, 8pu1_A, 8pu1_B, 8q66_A, 8q66_B, 8q6q_B, 8qc1_A, 8qc1_B, 8qci_A, 8ql1_A, 8qlk_A, 8r7t_A, 8raj_A, 8rd3_B, 8rgi_B, 8roc_A, 8roi_A, 8rp0_A, 8rsu_A, 8rz0_A, 8s02_A, 8s02_B, 8s1x_B, 8s2m_A, 8s2m_B, 8s3m_A, 8s6o_A, 8s6u_A, 8s7e_B, 8sao_B, 8sjj_A, 8suf_B, 8t5e_A, 8tj3_A, 8tk9_A, 8twd_A, 8tyx_A, 8tyx_B, 8u9o_B, 8uc6_A, 8ujh_A, 8upa_A, 8v3r_A, 8vk9_B, 8vrs_B, 8vvy_B, 8vx9_A, 8vx9_B, 8wee_A, 8whk_A, 8whk_B, 8wi2_A, 8wlj_A, 8wnt_B, 8wqr_A, 8wt0_A, 8wt0_B, 8wwc_A, 8wwy_B, 8x4s_A, 8x8t_B, 8xwy_A, 8xwy_B, 8yht_B, 8yhu_B, 8ytf_A, 8z27_B, 8za7_A, 8za7_B, 8zht_A, 8zht_B, 8ziw_A, 8znl_A, 8zpw_A, 8zrt_A, 8zue_B, 8zwe_A, 9atn_A, 9avf_B, 9b3h_A, 9b3h_B, 9b6m_A, 9b6m_B, 9biy_A, 9bk6_A, 9btm_A, 9c3b_A, 9c3b_B, 9c5t_A, 9cc5_A, 9cc6_A, 9cce_A, 9ch4_A, 9ci7_B, 9cqi_B, 9cu8_A, 9cys_B, 9d20_B, 9dgo_B, 9dir_A, 9dis_A, 9dk2_A, 9dk2_B, 9dpf_A, 9dsh_A, 9dz6_A, 9e1j_A, 9e1j_B, 9e6y_B, 9eby_A, 9ejw_B, 9eq6_B, 9erv_A, 9erv_B, 9f28_A, 9f28_B, 9f2d_A, 9f2j_B, 9f2m_A, 9fid_B, 9fnd_A, 9fnd_B, 9fqk_A, 9fqk_B, 9fun_A, 9fun_B, 9fyc_B, 9g6z_A, 9g7g_A, 9g87_A, 9g87_B, 9g8h_A, 9g8u_A, 9g8u_B, 9gbc_A, 9gbc_B, 9gei_A, 9gi0_B, 9gjc_A, 9gtz_B, 9gvh_B, 9h5r_A, 9h5r_B, 9h71_B, 9hac_A, 9hac_B, 9had_B, 9haf_B, 9hl2_B, 9i0e_A, 9igo_B, 9ild_A, 9ils_A, 9j2g_B, 9j4p_B, 9j5e_A, 9j9w_A, 9j9w_B, 9jbm_A, 9jdd_A, 9jdd_B, 9jdi_A, 9jdi_B, 9jm7_A, 9jm7_B, 9jm8_A, 9jm8_B, 9jqx_A, 9ju4_B, 9jvt_A, 9jw0_A, 9jwa_A, 9jwa_B, 9k1n_A, 9k1n_B, 9k3e_A, 9k3e_B, 9k9p_A, 9k9p_B, 9kp1_A, 9kp1_B, 9kqc_A, 9krb_B, 9l93_A, 9l93_B, 9leq_A, 9leq_B, 9lgw_A, 9lgw_B, 9lp2_A, 9lp2_B, 9ls0_A, 9ltp_A, 9lum_A, 9lv4_B, 9m3b_A, 9m3b_B, 9m4s_A, 9mey_B, 9mlu_B, 9mqb_A, 9n2f_A, 9n2f_B, 9n7y_A, 9nfu_B, 9nlt_B, 9nor_B, 9nu4_B, 9nv3_A, 9nv3_B, 9nzh_A, 9omd_A, 9omd_B, 9oq0_A, 9oq0_B, 9p3f_A, 9p3f_B, 9p3j_B, 9q1h_A, 9q1h_B, 9q1v_A, 9q79_A, 9q8o_B, 9q8z_B, 9qcd_B, 9qim_B, 9qqu_A, 9qu1_A, 9qu1_B, 9r2g_A, 9r4w_B, 9r85_B, 9r8g_A, 9r8g_B, 9rd0_B, 9rdc_A, 9rdc_B, 9rif_B, 9rsv_A, 9sf2_A, 9sqj_B, 9st8_A, 9st8_B, 9t3e_B, 9t3l_B, 9tb6_A, 9tpf_A, 9uap_A, 9uat_A, 9uf9_B, 9up2_A, 9upi_A, 9upz_A, 9v8i_A, 9v8i_B, 9vap_A, 9vap_B, 9ve6_A, 9vla_A, 9vll_A, 9vll_B, 9vno_A, 9vxg_A, 9wno_A, 9wno_B, 9x47_B, 9x8z_A, 9y5f_A, 9yj4_A, 9yj4_B, 9z5w_B, 9zm0_A.

TABLE S3 Separated heterodimeric chains ($n = 584$). PDB identifiers (with chain identifiers) of individual chains separated from heterodimeric complexes, used as negative examples for the homodimer classification task.

**Positive heterodimers**
7a67, 7acw, 7dnm, 7drb, 7e5z, 7eqx, 7et0, 7f28, 7f4t, 7fdo, 7ox5, 7q72, 7r49, 7rb3, 7rt7, 7s9v, 7tcr, 7tj4, 7vsi, 7wrs, 7xpc, 7y6b, 7y8u, 7yk3, 7yls, 7yn3, 7ytt, 7zhm, 8a82, 8arc, 8b0u, 8b4o, 8ba1, 8bgm, 8blw, 8boz, 8c47, 8cem, 8ct8, 8e51, 8ehe, 8enb, 8ey4, 8ezs, 8fex, 8fn9, 8g0k, 8g0p, 8gja, 8gkq, 8glc, 8gp6, 8gs1, 8gxe, 8hci, 8hf3, 8hfp, 8hm3, 8hsb, 8in9, 8ipr, 8ji9, 8jkz, 8k0h, 8k1l, 8ogi, 8onw, 8or7, 8orn, 8ows, 8p97, 8pdc, 8pfd, 8pq7, 8pu1, 8q66, 8qc1, 8s02, 8s2m, 8ssm, 8tyx, 8vx9, 8whk, 8wt0, 8xwy, 8z3f, 8za7, 8zht, 9b3h, 9b6m, 9c3b, 9dk2, 9e1j, 9erv, 9f28, 9fnd, 9fqk, 9fun, 9g87, 9g8u, 9gbc, 9gjc, 9h5r, 9hac, 9j9w, 9jdd, 9jdi, 9jm7, 9jm8, 9jwa, 9k1n, 9k3e, 9k9p, 9kp1, 9l93, 9leq, 9lgw, 9lp2, 9m3b, 9n2f, 9nv3, 9omd, 9oq0, 9p3f, 9p6h, 9q1h, 9q8z, 9qu1, 9r8g, 9rdc, 9st8, 9tpf, 9v8i, 9vap, 9ve6, 9vll, 9wno, 9y5f, 9yj4.

TABLE S4 Positive heterodimers (n = 139). PDB identifiers of the positive heterodimers used to compare the performance of heterodimer and homodimer prediction of AlphaFold2.3.

**Shuffled positive heterodimers**
22gu_A-8hnr_A, 22gu_A-8s89_A, 22oc_A-9gvh_B, 29sb_A-7vc7_A, 29sb_A-9jm8_A, 29sb_A-9lp2_A, 29sb_A-9m9g_A, 7b29_A-7pqw_A, 7b29_A-9jm8_A, 7djx_A-7fia_A, 7djx_A-9ded_A, 7djx_A-9lx1_A, 7dmf_A-9lm8_A, 7eet_A-9p6w_A, 7f5k_A-8uvv_A, 7fgz_A-9fw7_A, 7fgz_A-9kvu_A, 7fia_A-9gly_A, 7fia_A-9hsa_A, 7fia_A-9jwt_A, 7fia_A-9tpf_A, 7kuw_A-8zxg_A, 7ldf_A-8hnr_A, 7m5t_A-9ups_A, 7mcc_A-8agy_A, 7mcc_A-8qrx_A, 7mcc_A-9jm8_A, 7mn2_A-7mwq_A, 7mn2_A-7s9v_B, 7mn2_A-7uwy_A, 7mn2_A-8cws_B, 7mn2_A-8qrx_A, 7mwq_A-7fgz_A, 7mwq_A-9hsa_A, 7mwq_A-9m9g_A, 7osw_A-7djx_A, 7osw_A-8gac_A, 7osw_A-8gjg_A, 7osw_A-8y33_A, 7osw_A-9cic_A, 7osw_A-9dm3_A, 7pqw_A-7vgc_A, 7pqw_A-8ga7_A, 7pqw_A-9e9n_A, 7q58_A-7ver_A, 7qil_A-7osw_A, 7qil_A-7ue2_A, 7qil_A-9tpf_A, 7rmy_A-8iqs_B, 7s5l_A-8vc8_A, 7s5l_A-9lni_A, 7s9v_A-7s5l_A, 7s9v_A-9dcz_B, 7s9v_B-7ver_A, 7s9v_B-8ga7_A, 7s9v_B-8k7o_A, 7s9v_B-9i38_A, 7s9v_B-9y5f_A, 7t03_A-8qsx_A, 7t03_A-8znu_A, 7t03_A-9dsh_A, 7u61_A-7fia_A, 7u61_A-9dee_A, 7u61_A-9kgz_A, 7ur8_A-7vbq_B, 7ur8_A-8ju8_A, 7uwy_A-22oc_A, 7uwy_A-7vqv_A, 7uwy_A-7zb1_A, 7uwy_A-8qkd_A, 7uwy_A-8znu_A, 7uwy_A-9b6m_B, 7v8w_A-9gvh_B, 7v8w_A-9zdo_A, 7vbq_B-7kuw_A, 7vbq_B-7pw1_A, 7vc7_A-9szu_A, 7ver_A-8j3q_A, 7ver_A-9hsa_A, 7ver_A-9m3b_B, 7vfc_A-7s9v_A, 7vfc_A-8dj8_A, 7vfc_A-9fwa_A, 7vfc_A-9ups_A, 7vgc_A-7dmf_A, 7vgc_A-8uvv_A, 7vqv_A-8akp_A, 7y54_A-9jwt_A, 7y54_A-9m3b_A, 7y6b_A-8oys_A, 7yh8_A-7osw_A, 7yh8_A-9qs1_A, 7ypl_A-7t03_A, 7ypl_A-8qrx_A, 7ypl_A-8znu_A, 7ypl_A-9b6m_B, 7ypl_A-9gvh_B, 7zb1_A-9dd0_A, 8agy_A-7zb1_A, 8agy_A-9gvh_B, 8agy_A-9lni_A, 8akp_A-8j3q_A, 8cws_B-7vgc_A, 8dj8_A-7mcc_A, 8dj8_A-7mn2_A, 8dj8_A-9n8r_A, 8fg6_B-22oc_A, 8gjg_A-8akp_A, 8h2f_A-9b6m_A, 8h2f_A-9frq_A, 8h2f_A-9y5f_A, 8hnr_A-9p6w_A, 8iqs_B-8ah9_A, 8iqs_B-9jm8_A, 8iqs_B-9m9g_A, 8j3q_A-7kuw_A, 8j3q_A-7ue2_A, 8j3q_A-7yh8_A, 8j3q_A-8hnr_A, 8j3q_A-8rz0_A, 8j3q_A-9jm7_A, 8jcq_A-9fdg_A, 8jh8_A-22oc_A, 8jh8_A-9lni_A, 8ju8_A-8akp_A, 8k2j_A-8oys_A, 8k2j_A-8vez_A, 8k2j_A-9fwa_A, 8k2j_A-9hac_A, 8k7o_A-9i38_A, 8k7z_A-8vni_A, 8k8i_A-9m3b_B, 8oys_A-7u61_A, 8prj_A-7y6b_B, 8prj_A-9lp2_A, 8prj_A-9m3b_B, 8qkd_A-9gvh_B, 8qrx_A-8fg6_B, 8qrx_A-9rb0_A, 8qsx_A-7s9v_B, 8qsx_A-8qrx_A, 8qsx_A-8rz0_A, 8rz0_A-9tpf_A, 8s6c_A-8zxg_A, 8s6c_A-9ups_A, 8s89_A-8zxg_A, 8s89_A-9gvh_B, 8tw1_A-7kuw_A, 8tw1_A-7ver_A, 8tw1_A-8h2f_A, 8tw1_A-8k7z_A, 8tw1_A-8vc8_A, 8uvv_A-22gu_A, 8uvv_A-8znu_A, 8vez_A-7vc7_A, 8vez_A-9jm8_B, 8vez_A-9uml_A, 8vni_A-7mn2_A, 8vni_A-9n8r_A, 8vni_A-9v3a_A, 8x2v_A-7djx_A, 8x2v_A-8dj8_A, 8x2v_A-8ga7_A, 8znu_A-7s9v_B, 8znu_A-9x1x_A, 9avh_A-7m5t_A, 9b6m_B-8jh8_A, 9b6m_B-9fw5_A, 9b6m_B-9qs1_A, 9cic_A-7zb1_A, 9cic_A-9hsa_A, 9d01_A-7zb1_A, 9d01_A-8akp_A, 9d01_A-8y33_A, 9dcz_B-9tpe_B, 9dea_A-7vc7_A, 9ded_A-7y54_A, 9dee_A-7osw_A, 9dm3_A-7s9v_A, 9dsh_A-8iqs_B, 9dsh_A-8k2j_A, 9dsh_A-9fw7_A, 9e9n_A-7vfc_A, 9e9n_A-8bl3_A, 9exk_A-7vfc_A, 9f9r_A-7mwq_A, 9f9r_A-7rmy_A, 9f9r_A-7vqv_A, 9f9r_A-9fo1_A, 9f9r_A-9gly_A, 9fdg_A-7v8w_A, 9fo1_A-8znu_A, 9frq_A-9cic_A, 9fw7_A-8akp_A, 9fw7_A-9jm8_A, 9fwa_A-9vla_A, 9gvh_B-7zb1_A, 9gvh_B-8j3q_A, 9gvh_B-9dm3_A, 9gvh_B-9ukw_A, 9gvh_B-9uml_A, 9hac_A-8s6c_A, 9i38_A-7ur8_A, 9i38_A-9d01_A, 9jm7_A-8uvv_A, 9jm7_B-8k7z_A, 9jm7_B-9rb0_A, 9jm8_A-8agy_A, 9jm8_A-8gjg_A, 9jm8_A-8s6c_A, 9jm8_A-9hac_B, 9jm8_B-9jm7_A, 9jm8_B-9jm7_B, 9jm8_B-9lni_A, 9jm8_B-9tpf_A, 9kvn_A-8bl3_A, 9kvu_A-9ups_A, 9lm8_A-7kuw_A, 9lm8_A-8tw1_A, 9lm8_A-9fw7_A, 9lm8_A-9jm8_B, 9lni_A-9avh_A, 9lni_A-9jm8_A, 9lni_A-9lp2_A, 9lni_A-9tpf_A, 9lp2_A-7y6b_A, 9lp2_A-8akp_A, 9lp2_A-8qkd_A, 9lp2_B-9cic_A, 9lp2_B-9d01_A, 9lp2_B-9jm8_B, 9lx1_A-7vfc_A, 9lx1_A-9jwt_A, 9m3b_A-7ldf_A, 9m3b_A-8agy_A, 9m3b_A-8ah9_A, 9m3b_A-8j3q_A, 9m3b_A-9hac_A, 9m9g_A-8bl3_A, 9m9g_A-9lx1_A, 9m9g_A-9omb_A, 9n7y_A-7vbq_B, 9n7y_A-9b6m_B, 9n7y_A-9qs1_A, 9n8r_A-7uwy_A, 9n8r_A-9vla_A, 9o99_A-7ldf_A, 9o99_A-7vty_A, 9omb_A-29sb_A, 9omb_A-8ga7_A, 9omb_A-9b6m_A, 9omb_A-9frq_A, 9omb_A-9lx1_A, 9omb_A-9ups_A, 9p6w_A-9lp2_A, 9p6w_A-9lp2_B, 9qs1_A-22gu_A, 9qs1_A-7t03_A, 9qs1_A-9szu_A, 9qs1_A-9v4l_A, 9r8p_A-7fia_A, 9szu_A-7s9v_B, 9tpe_B-9n7y_A, 9tpf_A-7s5l_A, 9tpf_A-7ur8_A, 9tpf_A-9m9g_A, 9udh_A-7ver_A, 9udh_A-9fw5_A, 9ukw_A-9b6m_A, 9uml_A-7vbq_B, 9uml_A-9dsh_A, 9uml_A-9vsj_A, 9ups_A-7fia_A, 9ups_A-9fdg_A, 9ups_A-9m3b_B, 9ups_A-9n7y_A, 9v3a_A-7eet_A, 9v3a_A-7vbq_B, 9v3a_A-8h2f_A, 9v4l_A-7s9v_B, 9v4l_A-8k7o_A, 9v4l_A-9jm8_B, 9v4l_A-9ups_A, 9vla_A-8znu_A, 9vsj_A-9dcz_B, 9w4x_A-9n7y_A, 9x1x_A-8dj8_A, 9x1x_A-8oys_A, 9y5f_A-7ypl_A, 9y5g_B-9kvu_A, 9zdo_A-7mwq_A, 9zdo_A-9lx1_A, 9zdo_A-9omb_A.

TABLE S5 Negative heterodimers (n = 293). Identifiers of shuffled (recombined) positive heterodimer pairs and monomers, used as negative examples for the heterodimer classification task.

TABLE S6 Results of individual feature performance collected for homodimer performance improvement.

Feature	AUPR (↑)	F1@1%FPR (↑)	MCC@1%FPR (↑)	TPR@1%FPR (↑)	TPR@5%FPR (↑)
Min ipSAE	**0.80**	0.35	0.36	0.21	0.61
Max ipSAE	0.79	0.34	0.35	0.21	0.58
Average ipSAE	**0.80**	0.33	0.34	0.20	**0.62**
Max ipTM	0.79	0.36	0.37	0.23	0.59
Min ipTM	0.79	0.37	**0.38**	0.23	0.61
Average ipTM	**0.80**	0.37	0.37	0.23	0.61
Max pTM	0.78	0.34	0.35	0.21	0.55
Min pTM	0.79	0.36	0.37	0.23	0.61
Average pTM	0.79	0.36	0.37	0.22	0.61
Max ranking confidence	0.79	0.36	0.37	0.22	0.59
Min ranking confidence	**0.80**	0.37	**0.38**	0.23	0.61
Average ranking confidence	**0.80**	0.36	0.37	0.23	0.61
Multimer fraction (TM ≥0.0)	0.42	0.00	0.00	0.01	0.11
Multimer fraction (TM ≥0.1)	0.42	0.00	0.00	0.01	0.11
Multimer fraction (TM ≥0.2)	0.42	0.00	0.00	0.01	0.12
Multimer fraction (TM ≥0.3)	0.41	0.00	0.00	0.00	0.04
Multimer fraction (TM ≥0.4)	0.45	0.00	0.00	0.01	0.04
Multimer fraction (TM ≥0.5)	0.47	0.00	0.00	0.01	0.04
Multimer fraction (TM ≥0.6)	0.53	0.00	0.00	0.02	0.08
Multimer fraction (TM ≥0.7)	0.56	0.00	0.00	0.02	0.10
Multimer fraction (TM ≥0.8)	0.58	0.00	0.00	0.03	0.14
Multimer fraction (TM ≥0.9)	0.59	0.00	0.00	0.05	0.27
HM fraction (TM ≥0.0)	0.63	0.28	0.30	0.16	0.28
HM fraction (TM ≥0.1)	0.63	0.28	0.30	0.16	0.28
HM fraction (TM ≥0.2)	0.63	0.27	0.30	0.16	0.28
HM fraction (TM ≥0.3)	0.62	0.16	0.20	0.09	0.29
HM fraction (TM ≥0.4)	0.60	0.00	0.00	0.01	0.30
HM fraction (TM ≥0.5)	0.63	0.00	0.00	0.02	0.32
HM fraction (TM ≥0.6)	0.66	0.00	0.00	0.03	0.38
HM fraction (TM ≥0.7)	0.68	0.00	0.00	0.05	0.42
HM fraction (TM ≥0.8)	0.70	0.00	0.00	0.12	0.45

Feature	AUPR (↑)	F1@1%FPR (↑)	MCC@1%FPR (↑)	TPR@1%FPR (↑)	TPR@5%FPR (↑)
HM fraction (TM ≥0.9)	0.62	0.00	0.00	0.09	0.32
Best TM-score (all)	0.49	0.06	0.08	0.03	0.13
Best TM-score (multi)	0.54	0.09	0.12	0.05	0.17
Best TM-score (hm)	0.59	0.11	0.15	0.06	0.24
Contacts with max n models	0.69	0.15	0.19	0.08	0.34
Unique contacts	0.60	0.08	0.11	0.04	0.23
Mean contacts across predictions	0.75	0.29	0.32	0.17	0.47
Min contacts across predictions	0.78	0.31	0.33	0.19	0.54
Best num residue contacts	0.65	0.12	0.16	0.06	0.27
Best IF residues	0.65	0.14	0.18	0.08	0.27
Best pLDDT max	0.78	0.00	0.00	0.13	0.52
Best pAE min	0.39	0.00	0.00	0.00	0.01
Best Contact Score max	0.74	0.26	0.29	0.15	0.44
Buried apolar area (mean)	0.55	0.11	0.15	0.06	0.20
Buried polar area (mean)	0.47	0.06	0.07	0.03	0.15
Total interaction area (mean)	0.53	0.08	0.11	0.04	0.20
Frac buried apolar area (mean)	0.42	0.01	-0.01	0.01	0.05
Frac buried polar area (mean)	0.43	0.02	-0.01	0.01	0.05
Buried apolar area (min)	0.66	0.20	0.23	0.11	0.33
Buried polar area (min)	0.59	0.12	0.16	0.07	0.26
Total interaction area (min)	0.64	0.19	0.23	0.11	0.31
Frac buried apolar area (min)	0.42	0.01	-0.03	0.00	0.03
Frac buried polar area (min)	0.39	0.01	-0.03	0.00	0.02
Buried apolar area (max)	0.43	0.01	-0.02	0.01	0.09
Buried polar area (max)	0.38	0.01	-0.04	0.00	0.07
Total interaction area (max)	0.42	0.01	-0.03	0.00	0.08
Frac buried apolar area (max)	0.39	0.01	-0.02	0.01	0.03
Frac buried polar area (max)	0.42	0.02	0.01	0.01	0.06

Feature	AUPR (↑)	F1@1%FPR (↑)	MCC@1%FPR (↑)	TPR@1%FPR (↑)	TPR@5%FPR (↑)
Structural consensus (mean)	0.76	0.27	0.30	0.16	0.56
Structural consensus (min)	0.75	0.28	0.30	0.16	0.56
Structural consensus (max)	0.77	0.35	0.36	0.22	0.50
ipTM spread	0.65	0.33	0.35	0.20	0.36
rc spread	0.65	0.31	0.33	0.19	0.37
ipTM rc prod	0.79	**0.38**	**0.38**	**0.24**	0.61
ipSAE iptm diff	0.71	0.22	0.26	0.13	0.52
HM minus multimer TM==0.8	0.58	0.00	0.00	0.01	0.05
HM high any	0.65	0.00	0.00	0.02	0.11
HM low high gap	0.57	0.12	0.16	0.06	0.28
Foldseek specificity	0.55	0.00	0.00	0.00	0.31

TABLE S7 Explanation of each feature retrieved using code from SPOC. Taken from Table S3 of the supplementary material of Predictomes [16].

**Feature**	**Explanation**
Max ipTM	Highest ipTM of the 5 predicted models.
Min ipTM	Lowest ipTM of the 5 predicted models.
Mean ipTM	Average ipTM of the 5 predicted models.
Number of Contacts with Max N Models	Number of unique inter-residue pairs (contacts) found across interfaces in max_n_models (Max_n_models: The maximum number of times any unique contact appears across all models run for a given pair. Normalised by the number of predictions run to be between 0 and 1.).
Number of Unique Contacts	Number of unique inter-residue pairs (contacts) identified across all AF-M structural predictions for a given pair.
Mean Contacts Across Predictions	Mean number of contacts among all AF-M predictions for a given pair.
Min Contacts Across Predictions	Minimum number of residue pairs (contacts) found among all predictions for a given pair.
Best Num Residue Contacts	Number of residue pairs (contacts) in the interface of the single "best" structure prediction for a given pair.
Best IF Residues	Total number of residues involved in contacts in the interface of the single "best" structure prediction for a given pair.
Best pLDDT Max	Maximum pLDDT score of all interfacial residues of the single "best" structure prediction for a given pair.
Best pAE Min	Minimum PAE score of all interfacial residue pairs for the single "best" structure prediction for a given pair.
Best Contact Score Max	Maximum contact score for all residue pairs in the interface of the single "best" structure prediction for a given pair. The contact score is calculated as the number of atom contacts (dist < 5∀) between a pair of residues multiplied by their averaged pLDDT and divided by (1+ the average of the PAEs (x, y) and (y, x)).

TABLE S8 Results on an earlier version of the homodimeric dataset using different AlphaFold versions and QSProteome from[4]. AlphaFold2.0 was run using ColabFold[5].

Method	AUPR (↑)	F1@0.8 (↑)	MCC@0.8 (↑)	TPR@1%FPR (↑)	TPR@5%FPR (↑)
AF2.3 Max ranking confidence	0.78949	0.72616	0.60747	0.22067	0.58659
AF2.3 Min ranking confidence	0.79533	0.67698	0.59044	0.23464	0.61453
AF2.3 Average ranking confidence	0.79936	0.71240	**0.62071**	0.22626	0.61173
AF2.3 Max ipTM	0.78938	**0.72853**	0.61280	0.22626	0.59218
AF2.3 Min ipTM	0.79440	0.67817	0.59463	0.23045	0.61453
AF2.3 Average ipTM	0.79969	0.70500	0.61420	0.22765	0.61453
AF2.3 Max ipSAE	0.78709	0.68147	0.59348	0.20810	0.57682
AF2.3 Min ipSAE	**0.80332**	0.57524	0.51567	0.21369	0.60615
AF2.3 Average ipSAE	0.79661	0.62214	0.55241	0.20112	**0.62291**
AF3 Max ranking score	0.72935	0.64887	0.51907	0.15513	0.47263
AF3 Min ranking score	0.76192	0.61293	0.51728	**0.25850**	0.48764
AF3 Avg ranking score	0.75954	0.63221	0.52784	0.22814	0.47875
AF3 Max ipTM	0.76836	0.64041	0.53612	0.23154	0.49378
AF3 Min ipTM	0.77010	0.60000	0.51614	0.23034	0.51915
AF3 Avg ipTM	0.77329	0.61056	0.52084	0.22627	0.50468
AF3 Max ipSAE	0.76618	0.50704	0.46499	0.23464	0.50140
AF3 Min ipSAE	0.77409	0.42444	0.40833	0.22067	0.51397
AF3 Avg ipSAE	0.76939	0.47254	0.44370	0.22905	0.50698
ColabFold (AF2.0) Min ipTM*	0.78701	0.64889	0.57103	0.24283	0.59733
ColabFold (AF2.0) Average ipTM*	0.78963	0.69100	0.60385	0.22162	0.59327
ColabFold (AF2.0) Max ipTM*	0.77135	0.71581	0.59095	0.22863	0.54488
ColabFold (AF2.0) Min ipSAE*	0.79308	0.50302	0.45904	0.20950	0.61173
ColabFold (AF2.0) Average ipSAE*	0.78893	0.56734	0.50658	0.22486	0.60196
ColabFold (AF2.0) Max ipSAE*	0.77490	0.64505	0.55660	0.21788	0.54888
QSProteome Max PAE4Con3	0.75817	0.54195	0.47778	0.18017	0.53073
QSProteome Min PAE4Con3	0.79141	0.40998	0.40019	0.22905	0.60196
QSProteome Avg PAE4Con3	0.78232	0.48205	0.44715	0.22486	0.61313

*The original AlphaFold2.0 from DeepMind did not include an ipTM-score since it was only trained to predict monomers. The score in this table is from a ColabFold implementation of ipTM.

TABLE S9 Area under the precision-recall curve (AUPR) recomputed at hypothetical signal-to-noise (positive:negative) ratios of 1:4, 1:10, 1:100, 1:1000. Precision is reweighted analytically from the empirical, ratio-independent ROC curve. The best value for each row is marked in bold.

Ratio	AUPR Homodimers (↑)	AUPR Heterodimers (↑)
1:4	0.68	**0.91**
1:10	0.51	**0.87**
1:100	0.15	**0.71**
1:1000	0.04	**0.52**

TABLE S10 Foldseek structural homologs at TM-score ≥ 0.999 with MMseqs sequence identity (F-ident), excluding homomultimer targets. One row per unique query–target PDB pair, with the best TM-score kept. F-ident is the maximum sequence identity found by MMseqs for that PDB pair; -- indicates that the pair was not found in the MMseqs results.

Query	Target assembly	TM-score	F-ident	Stoichiometry
9htw	9i7w-assembly1	1.000	1.000	heteromultimer
7ban	7bao-assembly1	1.000	0.985	monomer
8eei	8eel-assembly2	1.000	1.000	monomer
9vlf	9l6j-assembly1	1.000	1.000	monomer
7y3w	7y3x-assembly1	1.000	0.997	monomer
7y3w	7y3x-assembly2	1.000	0.997	monomer
8eei	8eej-assembly1	1.000	0.997	monomer
8jyb	9ln2-assembly1	1.000	1.000	monomer
8eei	8een-assembly1	1.000	0.995	monomer
8jyb	9lnz-assembly1	1.000	1.000	monomer
8jyb	9ln2-assembly2	1.000	1.000	monomer
9grw	9gv9-assembly1	0.999	1.000	monomer
9rfy	9rg3-assembly2	0.999	1.000	monomer
9rfy	9rg5-assembly2	0.999	1.000	monomer
8eei	8eej-assembly2	0.999	0.997	monomer
8jyb	9lnz-assembly2	0.999	1.000	monomer
8eei	8eem-assembly1	0.999	1.000	monomer
9g0k	9fcs-assembly1	0.999	0.995	monomer
9g0k	9fcs-assembly2	0.999	0.995	monomer
9fj8	9fjz-assembly1	0.999	1.000	monomer
9rfy	9rg0-assembly2	0.999	1.000	monomer
9rfy	9rfz-assembly2	0.999	1.000	monomer
9rfy	9rg1-assembly2	0.999	1.000	monomer

S:3 | SUPPORTING INFORMATION

S:3.1 | Construction of the PPI dataset

We assembled a curated, non-redundant dataset of monomers, homodimers and heterodimers from structures deposited in the Protein Data Bank (PDB) after 30 September 2021 (72,511 entries) retrieved on the 4th of June 2026. Monomers and homodimers were processed through a shared pipeline, while heterodimers required a dedicated multi-assembly pipeline described separately below.

S:3.1.1 | Data Source

Protein Data Bank (PDB) identifiers released after the cutoff date (30th September 2021) were retrieved from RCSB in batches and merged into a single list of 72,511 IDs. For each entry, the stoichiometry (monomer, homodimer, heterodimer) was determined from a pre-computed stoichiometry cache derived from the full PDB.

S:3.1.2 | Monomer and Homodimer Pipeline

Step 1: Structure Parsing.
Only entries with a *single* biological assembly in the stoichiometry cache were considered for monomers and homodimers (entries with multiple assemblies were handled separately for heterodimers; see below). For each qualifying entry, the mmCIF file was parsed to extract the deposited (SEQRES) and resolved (ATOM) sequences, and the fractional length difference between the two was computed. Of 58,858 entries queued, 28,605 were saved; the remainder were skipped due to DNA/RNA chains, more than two chains in the SEQRES records, or parsing errors.

Step 2: SEQRES/ATOM difference filter.
Entries where the total SEQRES length differed from the total resolved (ATOM) length by $\geq 5\%$ were removed, retaining 12,745 entries across monomers and homodimers (Table S11).

TABLE S11 Filtering by SEQRES/ATOM length difference ($\geq 5\%$ threshold).

Class	Input	Removed	Kept
Monomer	14,753	6,885	7,868
Homodimer	9,899	5,022	4,877
**Total**	**24,652**	**11,907**	**12,745**

Step 3: Chain Length Filter.
Chains shorter than 50 or longer than 2,000 residues were excluded, removing an additional 403 entries and leaving 12,342 (Table S12).

Step 4: Cross-Dataset Redundancy Removal
To prevent data leakage, all passing sequences were searched against a database of PDB sequences deposited *before* 30 September 2021 using MMseqs2 `easy-search`[13]. Entries whose chains matched any pre-cutoff sequence at $> 90\%$ sequence identity were

removed, leaving 3,124 entries (Table S13).

Step 5: Resolution Filter.

Resolution values were retrieved from the RCSB REST API. Entries with resolution > 4.5 Å were excluded; NMR structures (no resolution value) were retained. After this step, 1,739 monomers and 1,378 homodimers remained (3,117 total).

Step 6: Internal Clustering.

Each class was clustered independently using MMseqs2 `easy-cluster` with parameters `-min-seq-id 0.3 -c 0.8 -cov-mode 0`. One representative per cluster was retained, yielding 979 monomers and 716 homodimers.

Step 7: Multimer Contamination Filter (monomers and heterodimers only)

To ensure that no monomer in the final set could also form a multimer, FASTA sequences for all 72,511 post-cutoff PDB IDs were downloaded from RCSB and clustered at 90% identity using MMseqs2 `easy-cluster` with parameters `-min-seq-id 0.9 -c 0.8 -cov-mode 0`. Monomers whose cluster also contained any homo-oligomer or hetero-oligomer were removed, reducing the monomer set from 979 to 847 entries.

The same was repeated for heterodimers, but only those in the same cluster as another homomultimer were removed.

S:3.1.3 | Heterodimer Pipeline

Because the single-assembly pipeline produced very few heterodimers, a dedicated pipeline was developed that accepts PDB entries with *multiple* biological assemblies, provided all assemblies agree on the same stoichiometry string (e.g. all report "heterodimer"). Assembly 1 was used as the representative; if absent, the first assembly found on disk was selected. This yielded 5,333 heterodimer candidates.

Subsequent filtering applied the same logic as the monomer/homodimer pipeline, with two adaptations for heterodimers. First, the SEQRES/ATOM difference threshold was relaxed to 20% to increase the number of saved heterodimers. Second, all filters were applied *per chain*: an entry was only discarded if *all* of its valid chains failed a criterion. A chain could therefore be flagged as invalid (e.g. too short) while the partner chain was retained; the entry's `keep_chain` annotation was updated accordingly. Additionally, heterodimers where chain A and chain B shared ≥ 90% sequence identity were removed before length filtering.

After these initial filtering steps, chains were clustered at 30% identity using MMseqs2 `easy-cluster`. The cluster representatives were analyzed to identify complete pairs (both chains A and B are cluster representatives), mutual cross-cluster pairs (chains from two different PDB entries cross-cluster, but together form a valid A+B representative pair), and

TABLE S12 Chain length filter (50–2,000 aa).

Class	Excluded	Too short	Too long
Homodimer	30	25	5
Monomer	373	355	18
**Total**	**403**	**380**	**23**

TABLE S13 Cross-dataset MMseqs2 filter (identity > 90%).

Class	Kept	Excluded
Homodimer	1,384	3,463
Monomer	1,740	5,755
**Total**	**3,124**	**9,218**

single-chain entries (only one chain survived the filters). Complete pairs and resolved mutual pairs were counted as positive heterodimer pairs; single-chain entries were retained but are not used as positive pairs. The clustering analysis yielded 139 positive heterodimer pairs and 630 individual chains in total (Table S14). This was done to avoid bias towards a certain class of proteins.

Lastly, the entire PDB was clustered with 90% sequence identity and any heterodimers that shared clusters with homodimers were removed as negatives for the homodimer classification part of the study. This resulted in 121 complete pairs and 346 individual chains, for a total of 599 entries (since every chain is its own entry for negatives), see Table S15.

TABLE S14 Heterodimer dataset after internal clustering.

Type	IDs	Individual chains
Complete pairs	129	258
Mutual cross-cluster pairs	10	20
Single chain (A only)	219	219
Single chain (B only)	133	133
**Total**	**491**	**630**

TABLE S15 Heterodimer dataset after homomultimer filtering.

Type	IDs	Individual chains
Complete pairs	121	242
Mutual cross-cluster pairs	7	14
Single chain (A only)	211	211
Single chain (B only)	132	132
**Total**	**471**	**599**

S:3.1.4 | Quality control

DNA/RNA Contamination

All entries were queried against the RCSB API to identify structures that co-crystallize with nucleic acid chains (`polymer_entity_count_DNA/RNA`). Eighteen monomers were identified and removed (reducing the set from 847 to 828), while no homodimers or heterodimers were affected. Note that the original SEQRES parsing step attempts to detect DNA/RNA chains via empty sequence records; however, short DNA sequences (e.g. `CTATTAGCCTCA`) share single-letter codes with amino acids and can bypass this check, necessitating the explicit API query.

Reinspection of the Negative Homodimeric Dataset

After the initial filtering was completed it was discovered that the version that was used for filtering away multimers for monomers and homomultimers for heterodimers was not updated to include recent entries (2021 and forward). After checking again against an updated version of the PDB the monomeric dataset was reduced from 828 to 783 entries. The heterodimeric negative dataset were in a similar fashion reduced from 121 to 119 complete pairs and 346 single chains (212 A_chains and 134 B_chains). The single-chains include the cross-mutual pairs.

S:3.1.5 | Final Dataset

After all filtering and quality control steps, the dataset comprises 783 monomers, 716 homodimers, 584 negative heterodimers entries and 139 complete negative heterodimeric pairs. Table S16).

TABLE S16 Final dataset composition.

Class	Entries
**Homodimer Dataset**	—
Monomers	783
Homodimers	716
Heterodimers used as negatives	—
Heterodimers (complete pairs)	119 (238 entries)
Heterodimers (single-chain)	346
Total neg heterodimers entries	238+119=584
**Heterodimer Dataset**	—
Heterodimers (positive pairs)	139
Heterodimers Shuffled (single-chain)	293
Total heterodimer entries	139+293=432

S:3.1.6 | Negative Pair Generation

Homodimer Negatives

Monomers and individual heterodimeric chains were presented as self-pairs (protein-X: protein-X) to serve as negative examples for the homodimer classification task. This resulted in 783 monomeric and 584 chains from heterodimers, i.e. in total 1,367 negatives.

Heterodimeric Negatives

The number of negative heterodimeric pairs was set to match the negative-to-positive ratio of the homodimer dataset (1,456/710 $\approx$ 2.05), giving a target of 289 negative pairs for 139 positives. After curating the dataset, a few negative samples were removed from the homodimeric dataset; however, this still maintained a close to 1:2 ratio between positives and negatives.

Candidate negative pairs were drawn from the same pool of 1,456 proteins (monomers and individual heterodimer chains). Same-species pairs (sharing the same NCBI taxonomy ID) were preferred, as these represent a harder and more biologically realistic negative example; cross-species pairs were used as a fallback when same-species candidates were insufficient.

Each candidate pair was subjected to the following filters:

(i) the two proteins must originate from different PDB entries;

(ii) the pair must not already appear in the positive set;

(iii) each protein must have at least five known functional associations in STRING[14] (combined score $\geq$ 100), ensuring that the absence of a reported interaction reflects a genuine lack of association rather than insufficient experimental coverage;

(iv) the pair must not have a functional association in STRING (combined score $\geq$ 100, functional network);

(v) the two proteins must not co-occur in any PDB structure (verified via the RCSB search API).

Prior to final selection, all passing candidates were sorted deterministically and shuffled with a fixed random seed (42) to ensure full reproducibility.

S:3.2 | Feature Selection and Logistic Regression Tuning

Feature selection was performed entirely on a pooled training set, defined by `split_json_030726.json`); the held-out test partition was loaded only in the final evaluation cell and was never used to rank features, choose a subset size, or tune a regularization strength.

S:3.2.1 | Combined Features

Eight features were derived from the raw AlphaFold/Foldseek columns prior to selection: the spread between the average and minimum ipTM and RC scores across predictions (`iptm_spread`, `rc_spread`), the product of minimum ipTM and minimum RC (`iptm_rc_prod`), the gap between average ipSAE and average ipTM (`ipsae_iptm_diff`), and four Foldseek-derived

homology features (hm_minus_multimer_tm08, hm_high_any, hm_low_high_gap, foldseek_specificity) contrasting the fraction of homomultimer and heteromultimer hits reaching a TM-score of 0.8. Identifier, label and potentially leaking columns (e.g. highest_match_*, stoich_*, and the missing_* imputation-flag columns) were excluded from the candidate pool.

S:3.2.2 | Homology-Feature Ablation

The pipeline was executed twice, toggling a flag (EXCLUDE_HOMOLOGY_FEATURES) that removes every Foldseek/homology column (prefixes multimer_frac_tm, hm_frac_tm, highest_tm_, and their four engineered derivatives) from the candidate pool before selection begins. Each pass wrote its full sweep of evaluated subsets to disk (cv_sweep_with_homology.pkl / cv_sweep_without_homology.pkl), allowing a "with-homology" and a "without-homology" model to be compared side by side in the final test-set cell without hand-copying feature lists between runs.

S:3.3 | Cross-Validation Protocol and Scoring Function

Every candidate feature subset, regardless of which strategy proposed it, was scored by the same routine (fit_and_eval_cv) to keep comparisons across strategies on equal footing. For a given subset, rows were standardised and passed through a scikit-learn Pipeline combining a StandardScaler and an L2-penalized LogisticRegression (solver="lbfgs", class_weight="balanced" to counteract label imbalance). Out-of-fold predicted probabilities were obtained with cross_val_predict under CV_FOLDS=5-fold StratifiedKFold (shuffle=True, fixed random_state); because the scaler sits inside the pipeline it is refit independently in every fold, so no scaling statistics leak across folds. A decision threshold was then chosen, on those same out-of-fold probabilities, to maximise the Matthews correlation coefficient (matthews_corrcoef) over 500 candidate cut-offs. From the resulting out-of-fold predictions and probabilities, six metrics were recorded: area under the precision–recall curve (AUPR, via precision_recall_curve + auc), ROC-AUC (roc_auc_score), MCC, F1, and true-positive rate at a fixed 1% and 5% false-positive rate (read off the ROC curve by linear interpolation).

S:3.3.1 | Choice of Regularization Strength C.

scikit-learn parameterizes the logistic-regression penalty by C, the inverse of the regularisation strength (small $C \Rightarrow$ strong shrinkage). C enters the pipeline in two distinct roles that were kept separate throughout:

- **Ranking C**: the regularization strength of the LogisticRegression estimator used *internally* by RFE, RFECV and forward selection to rank or score features while the subset is being built. Only two values were tried: the software default $C = 1$, and a data-driven C_{best} obtained once, on the full train feature matrix, via LogisticRegressionCV with $C \in$ np.logspace(-4, 4, 20), 5-fold CV and scoring="average_precision". Every RFE/RFECV/forward-selection strategy below was therefore run twice, once ranking with $C = 1$, once ranking with C_{best}, to check that the final subset was not an artefact of one particular ranking regularisation.
- **Evaluation C**: the regularization strength of the *final* classifier fit on a selected subset inside fit_and_eval_cv. Three modes were evaluated for every subset produced by every strategy: (i) C=1.0, the fixed default; (ii) C=BEST, the same C_{best} described above, fixed across all subsets; and (iii) C=subset-CV, in which LogisticRegressionCV is re-run *on that specific subset* (same C-grid, 5-fold CV, average_precision scoring) to pick a subset- specific C. This yields, for every (strategy, k) pair, three cross-validated performance rows rather than one, so that the eventual best subset is not confounded with a lucky choice of C.

S:3.3.2 | Feature-Selection Strategies

Six complementary strategies were used to propose candidate subsets; each was swept over subset size k (or, for RFECV, chose k internally), and every resulting subset was scored with `fit_and_eval_cv` as described above.

Strategy 1: Recursive Feature Elimination (RFE).

`sklearn.feature_selection.RFE` was fit once per ranking C (see above) with a `LogisticRegression` estimator and `step=1`: at each iteration the feature with the smallest absolute coefficient is dropped and the model is refit, until exactly k features remain. This was repeated for every k from 2 up to the number of candidate features, giving a full performance-vs-k curve for both ranking-C settings.

Strategy 2: Recursive Feature Elimination with Cross-Validation (RFECV).

`sklearn.feature_selection.RFECV` automates the choice of k: it repeats the RFE elimination path inside its own internal `StratifiedKFold(CV_FOLDS)` cross-validation (scoring `"average_precision"`, `min_features_to_select=2`) and returns the subset size that maximises the internal CV score. The resulting subset was then re-scored externally with `fit_and_eval_cv`, run once per ranking-C setting.

Strategy 3: L1-Regularized Logistic-Regression Path.

An $L1$-penalized `LogisticRegression` (`solver="liblinear"`) was fit for 30 values of C spaced log-uniformly over $[10^{-3}, 10^{2}]$. At each C, `sklearn.feature_selection.SelectFromModel` retained the features whose fitted coefficient was non-zero, producing subsets that shrink monotonically as C decreases. Duplicate subsets arising from adjacent C values were discarded, and every remaining unique subset was cross-validated.

Strategy 4: Univariate Filtering (SelectKBest).

`sklearn.feature_selection.SelectKBest` with the ANOVA F-statistic (`f_classif`) ranked each feature independently of all others by its univariate association with the label, and the top-k features were taken for every k in the sweep range. Because features are scored independently, this strategy ignores redundancy between correlated features, in contrast to Strategies 1–3 and 5–6.

Strategy 5: Forward Sequential Feature Selection.

A greedy forward search (implemented directly rather than via `SequentialFeatureSelector`, to expose the per-step CV score) started from an empty feature set and, at each step, evaluated every remaining candidate feature by adding it to the current set and scoring the resulting `StandardScaler` + `LogisticRegression` pipeline with `cross_val_score` under a 3-fold `StratifiedKFold` and `scoring="average_precision"`. The feature giving the largest mean CV score was permanently added, and the process repeated until the full sweep range of k was covered. As with RFE, this was run once with ranking $C = 1$ and once with ranking $C = C_{\text{best}}$.

Strategy 6: Tree-Based Importance Ranking.

An `sklearn.ensemble.ExtraTreesClassifier` (300 trees, `class_weight="balanced"`) was fit once on the full pooled feature matrix. Features were ranked by `feature_importances_` (mean impurity decrease across the ensemble), and the top-k features were taken for every k in the sweep range.

S:3.3.3 | Exhaustive Comparison and Subset Selection

Every (*strategy*, *k*) subset generated above; RFE × 2 ranking-*C* values, RFECV × 2, the L1 path, SelectKBest, forward selection × 2, and tree importance, was pooled into a single list and re-scored under all three evaluation-*C* modes (`C=1.0`, `C=BEST`, `C=subset-CV`), giving one cross-validated AUPR, MCC, ROC-AUC, F1, TPR@5%FPR and TPR@1%FPR per (strategy, *k*, eval-*C*) triple. The best subset was read off this table by two complementary criteria, the maximum CV TPR@1%FPR (ties broken by CV AUPR) and the maximum CV AUPR (ties broken by CV TPR@1%FPR).

The methods that produced the best combinations were forward selection and RFE. Forward selection was the best for both the logistic regression version without homology and the version with homology that produced the best CV AUPR.

S:3.3.4 | Held-Out Test-Set Evaluation

The held-out test partition was loaded for the first and only time in a dedicated final cell. Both cached sweeps (with- and without-homology) were read back from disk, and for each side the single best subset by CV AUPR and the single best subset by CV TPR@1%FPR were carried forward as candidates (deduplicated if the same subset won both criteria). Each candidate's regularisation strength was determined by matching it against the cached sweep table and selecting the eval-*C* mode that yielded the best CV AUPR for that exact feature set.

Candidates were then refit once on the full pooled train+val set and scored on the test set, alongside raw single-feature baselines (average/minimum/maximum ipTM, RC and ipSAE, used directly as a score with no fitted model, with the sign chosen to maximise AUPR on the pooled set). Differences between the best baseline, best without-homology, and best with-homology model were tested for statistical significance with the McNemar's statistical hypothesis test.
